# A Novel Prognostic Risk‐Scoring Model Based on RAS Gene‐Associated Cluster in Pediatric Acute Myeloid Leukemia

**DOI:** 10.1002/cam4.70716

**Published:** 2025-03-10

**Authors:** Cai‐Ju Luo, Yilimuguli Abudukeremu, Ming‐Liang Rao, Dun‐Hua Zhou, Jian‐Pei Fang, Yang Li, Lu‐Hong Xu

**Affiliations:** ^1^ Department of Pediatrics, Sun Yat‐Sen Memorial Hospital Sun Yat‐Sen University Guangzhou Guangdong China; ^2^ Key Laboratory of Malignant Tumor Gene Regulation and Target Therapy of Guangdong Higher Education Institutes Sun Yat‐Sen University Guangzhou Guangdong China; ^3^ Department of Pediatrics The First People's Hospital of Kashi Prefecture Kashi Xinjiang China

**Keywords:** acute myeloid leukemia, drug sensitivity, immune infiltration, prognosis, RAS gene

## Abstract

**Background:**

With the rapid development of diagnostic techniques and treatment strategies, there are notable improvements in pediatric acute myeloid leukemia (AML) prognosis. Nevertheless, the pathogenesis of AML remains largely unknown. This study aims to investigate the RAS pathway‐associated genes based on bioinformatics analysis, and investigate their underlying mechanisms in the initiation and progression of AML.

**Materials and Methods:**

The UCSC Xena database was the source of the training set data, while the GSE192638 dataset was the source of the validation set data. Children in the training set were split up into two groups according to RAS pathway‐associated genes, and then differentially expressed genes (DEGs) of them were screened. To discover prognosis‐related genes and develop a prognostic risk‐scoring model, we employed One‐way Cox and LASSO regression analysis. The performance of the model was assessed by an independent validation dataset. Survival analysis was performed using the Kaplan‐Meier (K‐M) curve. Furthermore, we investigated the association between the prognostic risk‐scoring model and the correlation between immune infiltration and drug sensitivity. The expression levels of genes associated with reverse transcription‐polymerase chain reaction were quantified.

**Results:**

We built a prognostic risk‐scoring model comprising 26 DEGs. Depending on the risk score, AML patients were split up into two groups: high‐ and low‐risk groups. Notably, compared with the survival time of patients in the high‐ risk group, that in the low‐risk group was substantially prolonged. Univariate (uniCox) as well as multivariate Cox (multiCox) regression analyses were carried out, demonstrating that the risk score emerged as a separate risk factor for prognosis. A nomogram that incorporates clinical factors and prognostic risk scores was proposed to increase the accuracy of survival rates estimation. Subsequent analyses revealed significant connections of the risk score with the immune infiltration and drug sensitivity. The experimental results demonstrated significantly elevated expression levels of GCSAML, MED12L, and TCF4 in AML samples compared to normal samples.

**Conclusion:**

The developed prognostic risk‐scoring model, along with the identified key risk genes, holds promise as candidate prognostic biomarkers and treatment targets for pediatric AML.

**Abbreviations**
AML: acute myeloid leukemia
BP: biological process
CC: cellular components
CDF: cumulative distribution function
CMML: chronic myelomonocytic leukemia
DCs: dendritic cells
FAB: French‐American‐British
GCSAML: germinal center‐associated signaling and motility‐like
GO: Gene Ontology
JMML: juvenile myelomonocytic leukemia
KEGG: Kyoto Encyclopedia of Genes and Genomes
K–M: Kaplan–Meier
MDS: myelodysplastic syndrome
MF: molecular function
OS: overall survival
PCA: principal component analysis
RNA‐seq: RNA sequencing
SD: standard deviation
ssGSEA: Single Sample Gene Set Enrichment Analysis
TCF4: transcription factor 4

## 1 Introduction

Acute myeloid leukemia (AML) is a group of malignant clonal diseases derived from myeloid stem and progenitor cells with high clinical and biological heterogeneity. It is characterized by a challenging prognosis and remains a significant cause of mortality among children and individuals under 35 years old in China. In the last few decades, there have been impressive breakthroughs in the prognosis of AML due to the rapid progress in diagnostic techniques and treatment strategies, and the overall long‐term survival stands at approximately 70% [1]. The pathogenesis of AML remains unclear, and the distortion of signaling pathways (such as FLT3 and RAS family members) is considered one of the leading pathogenic factors of AML.

RAS is a proto‐oncogene belonging to the small GTPase family, which comprises three subtypes such as NRAS, KRAS, and HRAS. It mainly participates in intracellular signal transduction [2]. Certain mutations in RAS genes result in the activation [2, 3, 4, 5, 6] of RAS signaling pathway by preventing the hydrolysis of GTP and maintaining RAS in a constitutively activated state. This type of mutation is common in various human malignant tumors, involving AML, chronic myelomonocytic leukemia (CMML), myelodysplastic syndrome (MDS), and juvenile myelomonocytic leukemia (JMML). The activation of RAS will lead to an increase in signaling pathways through two main pathways: the RAS/RAF/MEK and the RAS/PI3K pathways [7]. The activation is associated with enhanced proliferation of hematopoietic progenitor cells. In transience mouse models, oncogenic RAS mutations facilitate the development of myeloid phenotypes that closely resemble human CMML or AML [8, 9]. Several genes associated with the RAS pathway, involving NF1, PTPN11, and NRAS, have also been found to exhibit prognostic significance in pediatric AML [10]. It can be concluded that RAS pathway genes have important value in AML, but studies on prognostic biomarkers based on RAS pathway genes remain insufficient.

Here, we integrated RAS pathway‐associated genes, performed an extensive transcriptome analysis of pediatric AML utilizing bioinformatics approaches, and identified DEGs based on the RAS pathway. Subsequently, a prognostic risk model was developed, which exhibited high efficacy in anticipating the prognosis of pediatric patients with AML. The performance of the predictive model was validated by a series of analyses and verifications. The association of risk models with immune infiltration as well as chemotherapy response was further investigated. Additionally, the expression levels of different risk genes in primary AML samples were assessed. Our research findings may offer a novel and precise approach that holds great promise for the diagnosis and treatment of pediatric AML.

## 2 Materials and Methods

**FIGURE 1:**
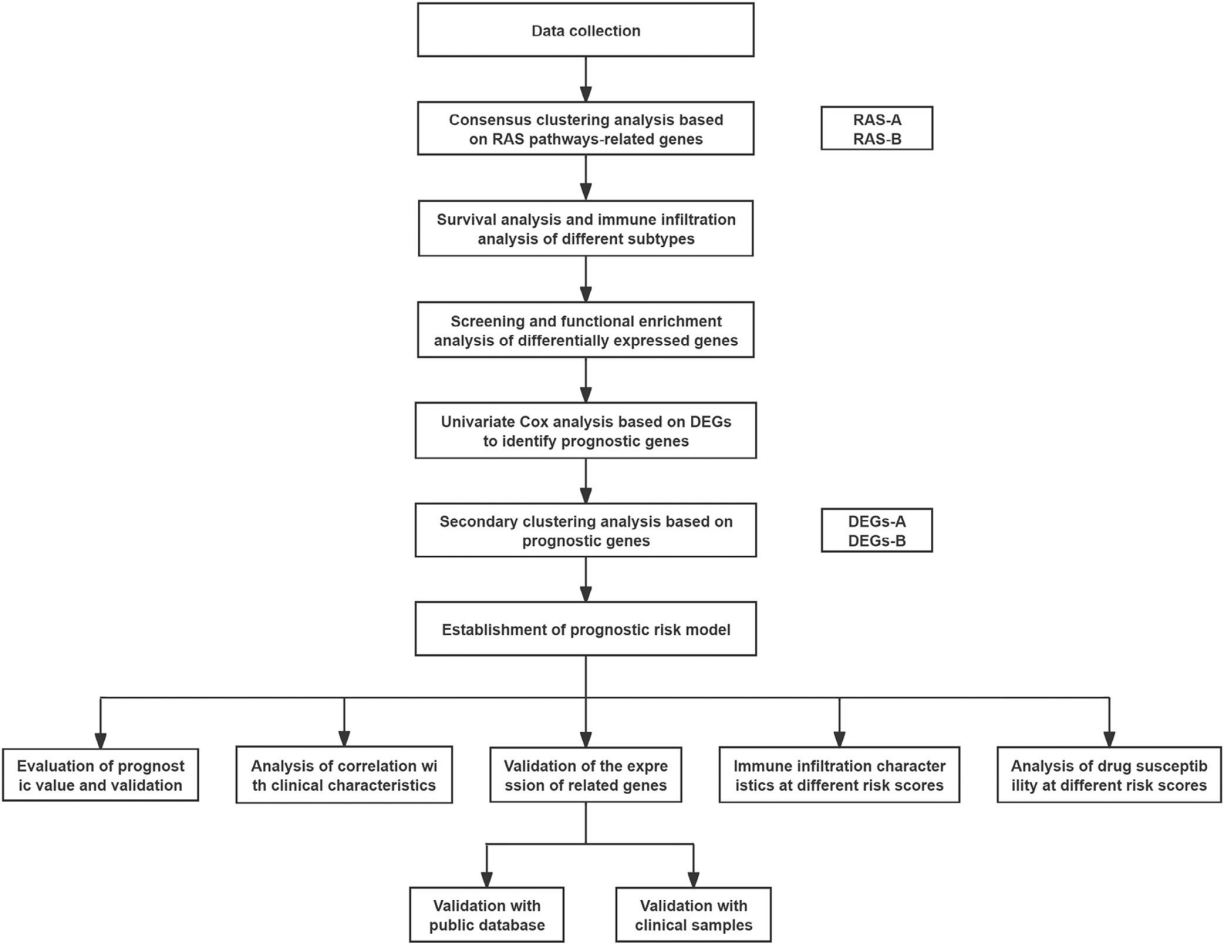
Flow diagram of the comprehensive study design.

The comprehensive experimental design is depicted in Figure 1.

### 2.1 Data Collection

The training set was sourced from RNA sequencing (RNA‐seq) data and pertinent clinical details (the original data were sourced from TARGET data) of 179 children with AML (age at diagnosis was under 18 years) collected from the UCSC Xena database (http://xena.ucsc.edu/), and these data were carefully chosen and collected for analysis. For the validation set, we utilized the GSE192638 dataset, consisting of 37 samples of AML patients gathered from the GEO database (https://www.ncbi.nlm.nih.gov/geo/). The clinical details of AML patients in TARGET and GEO cohorts are presented in Table 1. The RNA‐seq data of 179 children with AML were described by matrix using the “tinyarray” R package. Altogether, 236 genes associated with RAS pathway were acquired from the official website of KEGG (https://www.kegg.jp/).

**TABLE 1:** Information on AML patients in the training set and validation set.

Feature	Training set (*n* = 179)	Validation set (*n* = 37)
	No. (%) or Mean ± SD	No. (%) or Mean ± SD
Gender		
Male	86 (48.0%)	21 (56.8%)
Female	93 (52.0%)	16 (43.2%)
Age (years)	8.32 ± 5.69	8.55 ± 5.85
WBC at diagnosis (10^9^/L)	75.43 ± 94.63	51.87 ± 69.29
Bone marrow blast (%)	71.24 ± 20.86	76.42 ± 11.32
Chromosome karyotype analysis		
Normal karyotype	38 (21.2%)	5 (13.5%)
Complex karyotype	48 (26.8%)	19 (51.4%)
Other karyotype analyses	85 (47.5%)	13 (35.1%)
Unknown	8 (4.5%)	0 (0.0%)
Risk stratification		
Favorable	70 (39.1%)	10 (27.0%)
Intermediate	87 (48.6%)	15 (40.5%)
Adverse	12 (6.7%)	12 (32.4%)
Unknown	10 (5.6%)	0 (0.0%)
Survival status		
Alive	89 (49.7%)	19 (51.4%)
Death	90 (50.3%)	18 (48.6%)

### 2.2 Consistency Clustering Analysis Based on RAS Pathway‐Associated Genes

Based on 236 RAS pathway‐associated genes, consistency clustering analysis was performed on 179 samples of AML patients using the “ConsensusClusterPlus” R package. Unsupervised analysis incorporated stability evidence to determine cluster counts and membership. The entire process was iterated 1000 times to guarantee the stability of the clustering results. The “survival” and “survminer” R packages were employed to generate Kaplan–Meier (K–M) overall survival curves among different clusters. The sample clustering was evaluated using the principal component analysis (PCA). The CIBERSORT algorithm was adopted for immune infiltration analysis, so as to assess the expression of different clusters on 22 types of immune cells.

### 2.3 Screening and Functional Enrichment Analysis of DEGs

The “limma” R package was adopted to screen DEGs among different subgroups, with a cutoff for log2 (fold change) exceeding 0.8 and a *p*‐value no more than 0.05. Moreover, the “ggplot2” R package was employed to illustrate a volcano map of DEGs, and the “pheatmap” R package was employed to create a heat map of DEGs. Gene Ontology (GO) and Kyoto Encyclopedia of Genes and Genomes (KEGG) were conducted using the “clusterProfiler” R package for DEGs. GO analysis primarily focused on biological process (BP), cellular components (CC), and molecular function (MF). Differences were determined to be statistically evident at *p* < 0.05 in G0 and KEGG analyses.

### 2.4 Development and Validation of Prognostic Risk‐Scoring Model

We conducted a uniCox regression analysis on DEGs to filter prognostic‐related genes. Subsequently, LASSO regression analysis was conducted with the “glmnet” R package to screen hub genes and create a prognostic risk‐scoring model. We calculated the risk score by taking into account the gene expression level and the regression coefficient, and the equation is as follows: Risk score = $\sum_{i}^{n}X_{i}Y_{i}$ (*X* represents the expression level of risk genes, *Y* represents the regression coefficient of risk genes). Depending on the median risk score, patients were assigned into two groups: high‐ and low‐risk groups. To assess the predictive accuracy of 1‐, 3‐, and 5‐year survival, the “survival” R package was used to conduct ROC curve analysis. Risk score calculation, risk stratification, survival outcome assessment, and ROC curve analysis on the GSE192638 dataset were conducted with the same method, so as to verify the consistency of models.

### 2.5 Correlation Evaluation of Clinical Characteristics, Survival Outcomes, and Risk Score

To further examine the correlation of clinical characteristics with the prognosis in AML patients, the clinical data were extracted from the training set of patients, involving sex, age, leucocyte count at the first visit, percentage of myeloid leukemia cells, percentage of primitive cells in peripheral blood, TAB typing, and risk score obtained from uniCox and multiCox regression investigation, so as to investigate if the risk score acts as a separate risk factor. However, a box plot was created to examine the correspondence of risk scores with disease risk stratification. A nomogram was generated using the “rms” R package to visually represent the correlation of clinical features with the prognostic risk‐scoring model. The scores of each prognostic factor were aggregated, allowing for the prediction of the 1‐, 3‐, and 5‐year survival of patients with AML according to the total score. In addition, we combined the previous grading results of AML patients with traditional risk stratification, risk scores, and survival outcomes, and used the sankey diagram to visualize the differences in scores among different grades to verify the accuracy of survival analysis results among different grades.

### 2.6 Analysis of Immune Cell Abundance

The mRNA expression profile of the training set was utilized, and the “ESTIMATE” R package was employed along with the ESTIMATE algorithm. We aimed to figure out and compare the stromal score, immune score, and ESTIMATE scores between two distinct groups: high‐ and low‐risk groups. To assess the abundance of infiltrating immune cells within each sample, the Single Sample Gene Set Enrichment Analysis (ssGSEA) was employed, so as to review variations in immune cell composition within the bone marrow microenvironment between two distinct groups. Afterward, the “pheatmap” R package was exploited to display the interaction of 26 prognostic genes and the connection of prognostic genes with immune cell infiltration.

### 2.7 Drug Sensitivity Analysis

The “oncoPredict” R package was utilized to figure out and compare the half‐maximal inhibitory concentration (IC50) values of rapamycin, sorafenib, dasatinib, cytarabine, venetoclax, and RAS inhibitors in different risk scores and to explore the differences in drug sensitivity among AML patients with different scores.

### 2.8 Quantitative Real‐Time PCR (qRT‐PCR)

AML clinical samples were sourced from the specimen bank in our hospital. The collection of clinical information was conducted following the principles stated in the Declaration of Helsinki, ensuring the informed consent of both the patient and their family members. This study was granted ethical approval from the Ethics Committee of SUN Yat‐Sen Memorial Hospital, SUN Yat‐Sen University. The diagnosis of the child was determined using the French–American–British (FAB) classification systems. The clinical characteristics of AML patients are shown in Table 2. RNA was extracted by complying the instructions in SteadyPure RNA Rapid Extraction Kit (AG, China). The Evo M‐MLV reverse transcription reagent premix (AG, China) kit was applied for reverse transcription cDNA generation. qRT‐PCR was performed according to the instructions in NovoStart Probe qPCR SuperMix kit (Novoprotein, China). ACTB was chosen as the reference gene. The primers for GCSAML, MED12L, and TCF4 were as follows: GCSAML forward primer (5′‐TGCGAAAACTCAGGCAGGAA‐3′), reverse primer (5′‐AGAACCACTGCCATTCTCGT‐3′); MED12L forward primer (5′‐CAGACTCGGCCTTTCCAACA‐3′), reverse primer (5′‐CTGTGGCATGGTCTGTGCTT‐3′); GCSAML forward primer (5′‐CCTGGCTATGCAGGAATGTT‐3′), reverse primer (5′‐CAGGAGGCGTACAGGAAGAG‐3′). Graphpad Prism was applied for plotting and statistical analysis.

**TABLE 2:** Clinical characteristics of 10 children with AML.

Feature	Clinical samples (*n* = 10)
	No. (%) or Mean ± SD
Gender	
Male	8 (80.0%)
Female	2 (20.0%)
Age (years)	9.70 ± 4.34
WBC at diagnosis (10^9^/L)	35.61 ± 32.96
Hb at diagnosis (g/L)	85.50 ± 17.42
PLT at diagnosis (10^9^/L)	65.60 ± 40.51
Bone marrow blasts (%)	61.40 ± 24.51
Peripheral blasts (%)	39.60 ± 32.73
FAB category	
M1	2 (20.0%)
M2	1 (10.0%)
M4	2 (20.0%)
M5	4 (40.0%)
M7	1 (10.0%)
Chromosome karyotype analysis	
Normal karyotype	4 (40.0%)
Complex karyotype	4 (40.0%)
Other karyotype analyses	2 (20.0%)
Risk stratification	
Favorable	1 (10.0%)
Intermediate	5 (50.0%)
Adverse	4 (40.0%)
Survival status	
Alive	9 (90.0%)
Death	1 (10.0%)

### 2.9 Statistical Analysis

The entire data employed in this research were formulated as mean ± standard deviation (SD). The R software (V4.3.1) was utilized for all bioinformatics analyses. Additionally, the statistical analysis was conducted through Graphpad Prism. In the case of comparisons between two groups, the Wilcoxon test was employed, while in situations where more than two groups are involved in comparisons, the Kruskal–Wallis test is typically selected. Correlation analysis was performed with the Spearman test. Statistical significance was defined at *p* < 0.05, with * implying *p* < 0.05, ** indicating *p* < 0.01, and *** representing *p* < 0.001.

## 3 Results

### 3.1 Identification of RAS Subtypes in AML Patients

**FIGURE 2:**
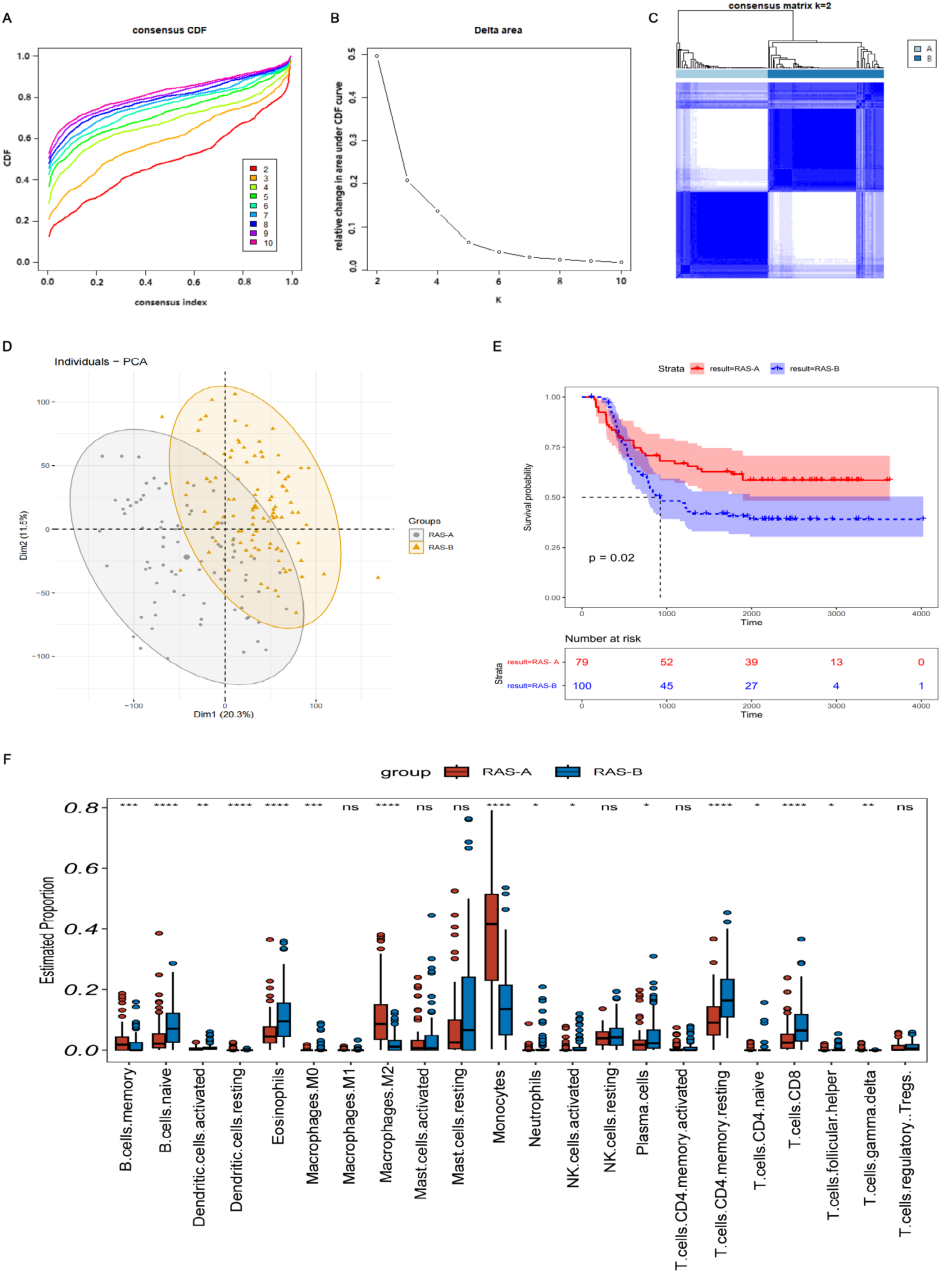
Classification of AML patients using RAS pathway‐associated genes. (A, B) Consensus CDF curve. (C) Optimal classification of AML through the consensus matrix (*k* = 2). (D) The principal component analysis graph showed the separation of two subgroups. (E) Survival analysis graph for two subgroups. (F) Abundance of immune infiltration in two subgroups.

RNA‐seq data and clinical details were obtained from a cohort of 179 patients with AML from the TCGA data. The expression profiles of 236 RAS genes were used for consistent clustering analysis. In light of the consensus cumulative distribution function (CDF) curve (Figure 2A,B), AML patients were divided into two subgroups, the RAS‐A subgroup and the RAS‐B subgroup (Figure 2C). PCA analysis revealed significant disparities in the expression of RAS genes between the two subgroups (Figure 2D). We further conducted survival analysis for the two subgroups. According to the K–M curve results, AML patients belonging to the RAS‐A subgroup exhibited a more favorable prognosis (overall survival [OS]) than those belonging to the RAS‐B subgroup (*p* = 0.02, Figure 2E). RAS genes are involved in the regulation of recruitment, activation, and differentiation of immune cells to coordinate tumor cells to evade immune surveillance [11, 12]. Further comparison on the immune infiltration characteristics was made between the two subgroups, and the findings from this research demonstrated substantial disparities in the infiltration of multiple immune cells between the two groups, mainly in B‐cell naive, resting dendritic cells (DCs), eosinophils, macrophages M2, monocytes, resting memory CD4 T cells, and CD8 T cells (Figure 2F).

### 3.2 Identification and Enrichment Analysis of DEGs

**FIGURE 3:**
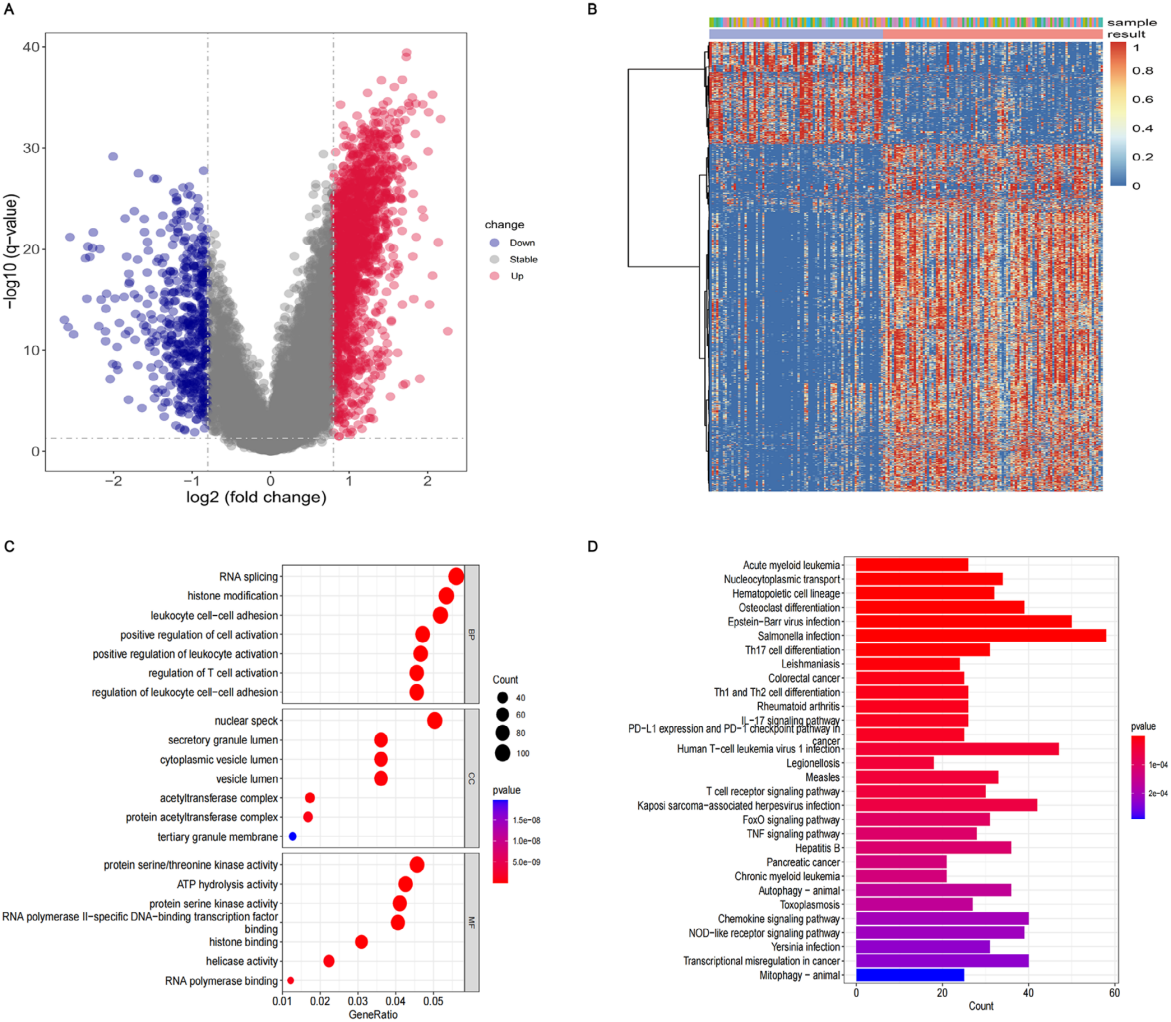
Identification and functional enrichment analysis of DEGs between two subgroups. (A) Volcano plot of DEGs. (B) The expression levels of two subgroups of DEGs showing by the heat map. (C) GO enrichment analysis results. (D) KEGG enrichment analysis results.

Altogether, 2203 DEGs were identified between the two subgroups, with 1700 genes showing upregulation and 503 genes showing downregulation (Figure 3A). Additionally, the heat map of 2203 DEGs was plotted (Figure 3B), and the results disclosed substantial differences in DEGs between subgroups A and B. GO and KEGG pathway enrichment analyses were conducted in an effort to further investigate the potential biological function of DEGs. The GO analysis results revealed notable enrichment of DEGs in various BPs, including RNA splicing, histone modification, leukocyte‐cell adhesion, and upregulation of cell activation. The CC was enriched in nuclear speckles, secretory granule cavities, and cytoplasmic vesicles. The MF was enriched in protein serine/threonine kinase activity, ATP hydrolytic activity, and protein serine kinase activity (Figure 3C). Enrichment analysis of the KEGG pathways revealed significant associations with AML, cancer, and infections (Figure 3D).

### 3.3 The Development and Validation of Prognostic Risk‐Scoring Model Depending on RAS Pathway Genes

**FIGURE 4:**
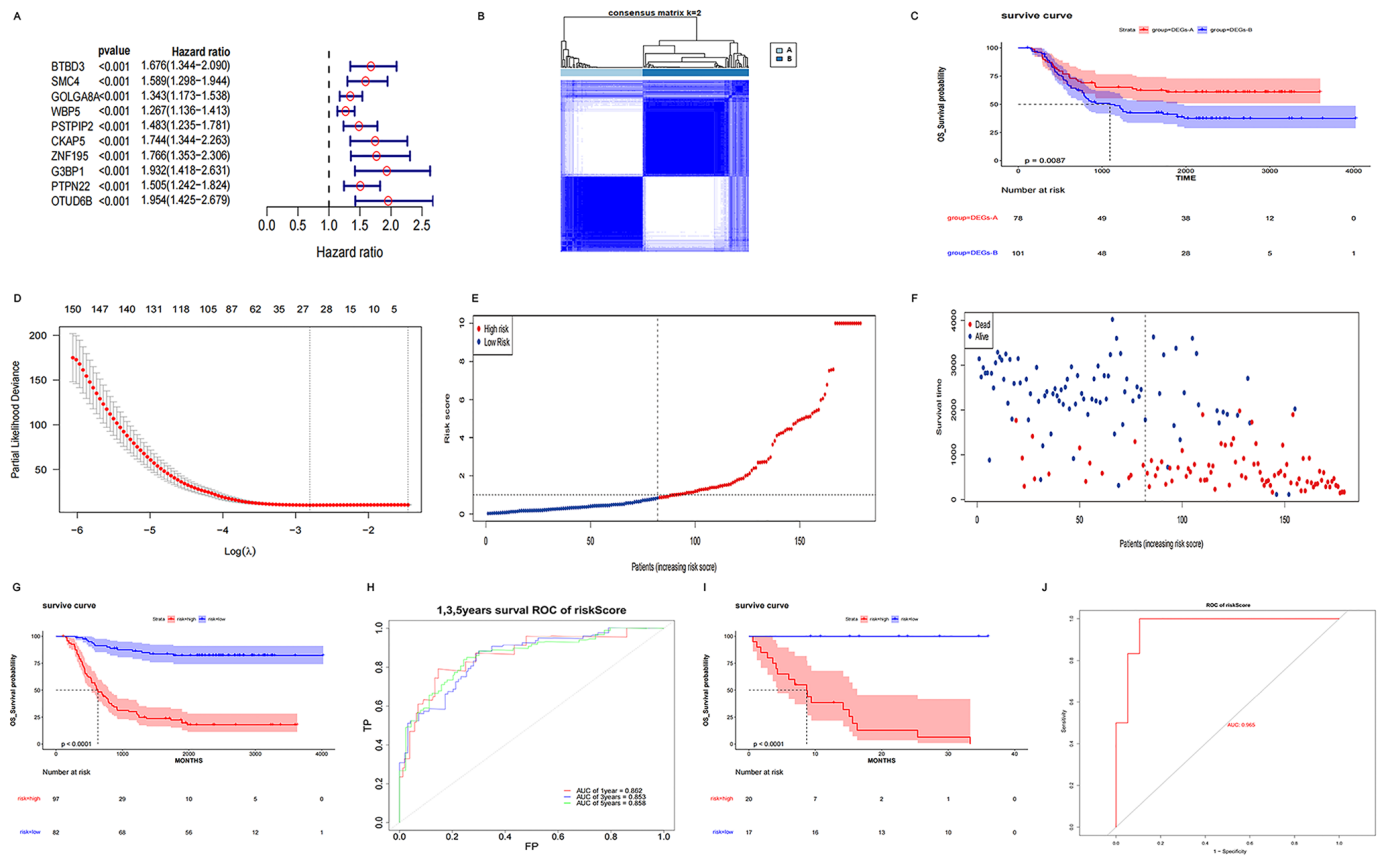
The development and validation of prognostic risk‐scoring model depending on 26 genes. (A) A forest plot depicting the prognostic significance of the top 10 RAS pathway‐associated genes was generated through uniCox regression analysis. (B) AML was tested again using the consensus matrix (*k* = 2). (C) Survival analysis of two gene clusters. (D) LASSO regression cross‐validation. (E) Distribution of risk scores in AML patients. (F) Survival outcomes of AML patients with distinct risk scores. (G) Survival analysis of risk‐stratified groups in the training cohort. (H) ROC curve of the training set. (I) Survival analysis of risk‐stratified groups in the validation cohort. (J) ROC curve of the validation set.

DEGs were analyzed with uniCox regression to identify prognostic genes, where BTBD3, SMC4, GOLGA8A, WBP5, PSTPIP2, CKAP5, ZnF195, G3BP1, PTPN22, and OTUD6B were the top 10 genes associated with prognosis (Figure 4A). Unsupervised clustering analysis was performed on the prognostic genes to sort patients into two genomic subtypes (Figure 4B). As reported by the K–M curve analysis, patients belonging to the DEGs‐A cluster exhibited markedly prolonged OS compared to those in the DEGs‐B cluster (*p* = 0.0087, Figure 4C).

Lasso cox regression analysis was utilized to refine the candidate gene pool and construct an accurate prognostic risk‐scoring model (Figure 4D), and 26 genes (OTUD6B, ABHD13, BTBD3, GSTK1, WT1, METTL7B, NUDT1, DCPS, BCKDK, N4BP3, DGKE, GCSAML, TCF4, KCNK5, SH2D1A, MED12L, CTSD, CD300LF, TMEM176A, SLC7A7, MSLN, MT1X, CD1C, TNFSF13B, SELL, CCL2) were ultimately selected to establish the prognostic model. Lasso cox analysis was used to figure out the risk score for each patient with AML, and all patients were classified into two groups: high‐ and low‐risk groups (Figure 4E). By comparing with the children in the high‐risk group, those in the low‐risk group had a longer life expectancy (Figure 4F). The impact of 26 genes on the prognosis was also analyzed. It was found that the genes with good prognosis included CD1C, GSTK1, MSLN, SLC7A7, SELL, and TMEM176A. The genes with poor prognosis included OTUD6B, ABHD13, BTBD3, WT1, METTL7B, NUDT1, DCPS, BCKDK, N4BP3, DGKE, GCSAML, TCF4, KCNK5, SH2D1A, MED12L, CTSD, CD300LF, MT1X, TNFSF13B, and CCL2 (Figure S1).

Survival analysis was performed on both high‐ and low‐risk groups, and it was found that, compared with children in the high‐risk group, those in the low‐risk group exhibited markedly extended OS, as indicated by the K–M survival curve analysis (*p* < 0.0001, Figure 4G). The ROC curve revealed the AUC values for the 1‐, 3‐, and 5‐year survival prognostic models as 0.862, 0.853, and 0.858, respectively (Figure 4H). To verify the predictive capability of the model, data from the GSE192638 cohort were calculated for prognostic analysis. The results manifested that the prognosis of AML patients in the low‐risk group was significantly better than that of the high‐risk group (*p* < 0.0001, Figure 4I). Considering the small sample size, the ROC curve of the risk score was calculated, with an AUC value of 0.965 (Figure 4J). It could be concluded that the consistency of predictive validity of our risk model was verified, and the RAS pathway‐based risk‐scoring model exhibited strong predictive performance in the test cohort.

### 3.4 Establishment of Clinical Characteristics and Prognostic Nomograms

**FIGURE 5:**
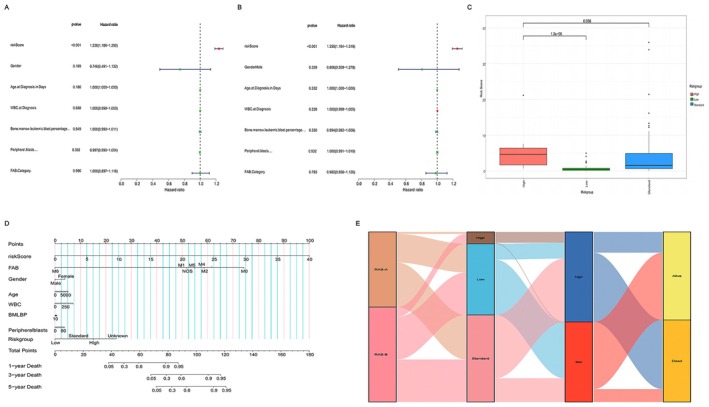
Correlation of clinical characteristics, risk scores, and prognostic significance in AML patients. (A) The uniCox analysis of clinical characteristics and risk scores. (B) The multiCox analysis of clinical characteristics and risk scores. (C) Correlation between disease risk stratification and risk scores. (D) Nomogram for anticipating 1‐, 3‐, and 5‐year survival in patients with AML. (E) Sankey diagram for presenting the distribution of RAS clusters, DEG clusters, risk scoring groups, and survival status of patients.

To comprehensively assess the clinical predictive value of the prognostic risk‐scoring model, we conducted uniCox and multiCox regression analyses, considering gender, age, white blood cell count at the first visit, percentage of myeloid leukemia cells, percentage of primitive cells in peripheral blood, TAB typing, and risk score as covariates. The uniCox regression analysis results disclosed that the risk score is meaningfully associated with the prognosis of individuals with AML (*p* < 0.001, Figure 5A). Moreover, multiCox regression analysis reported that the risk score was a separate prognostic factor in children with AML (*p* < 0.001, Figure 5B). Furthermore, we explored the association of risk scores with disease risk stratification. Significant variations in risk scores were observed across different risk subtypes, with the high‐risk group exhibiting an escalated median risk score compared to the low‐ and medium‐risk groups (Figure 5C). In clinical practice, children in the high‐risk group exhibited a relatively unfavorable prognosis, demonstrating consistency in the predictive effect. Therefore, the risk score could be served as a predictive indicator for prognosis. A nomogram (Figure 5D) was constructed incorporating a prognostic risk‐scoring model and several clinical features, based upon the findings from uniCox and multiCox regression analyses. The distribution of RAS clusters, traditional risk stratification, risk scoring groups, and survival outcome of each individual was presented in the sankey (Figure 5E). Most RAS‐B and traditional high‐risk (traditional risk stratification) groups with poor prognosis were considered as high‐risk groups, while the RAS‐A and traditional low‐risk groups with favorable prognosis tended to be in the low‐risk groups. Interestingly, two‐thirds of the patients in the traditional medium‐risk group were considered to have a high risk, and the remaining patients were classified as low risk.

### 3.5 Analysis of Tumor Immune Microenvironment

**FIGURE 6:**
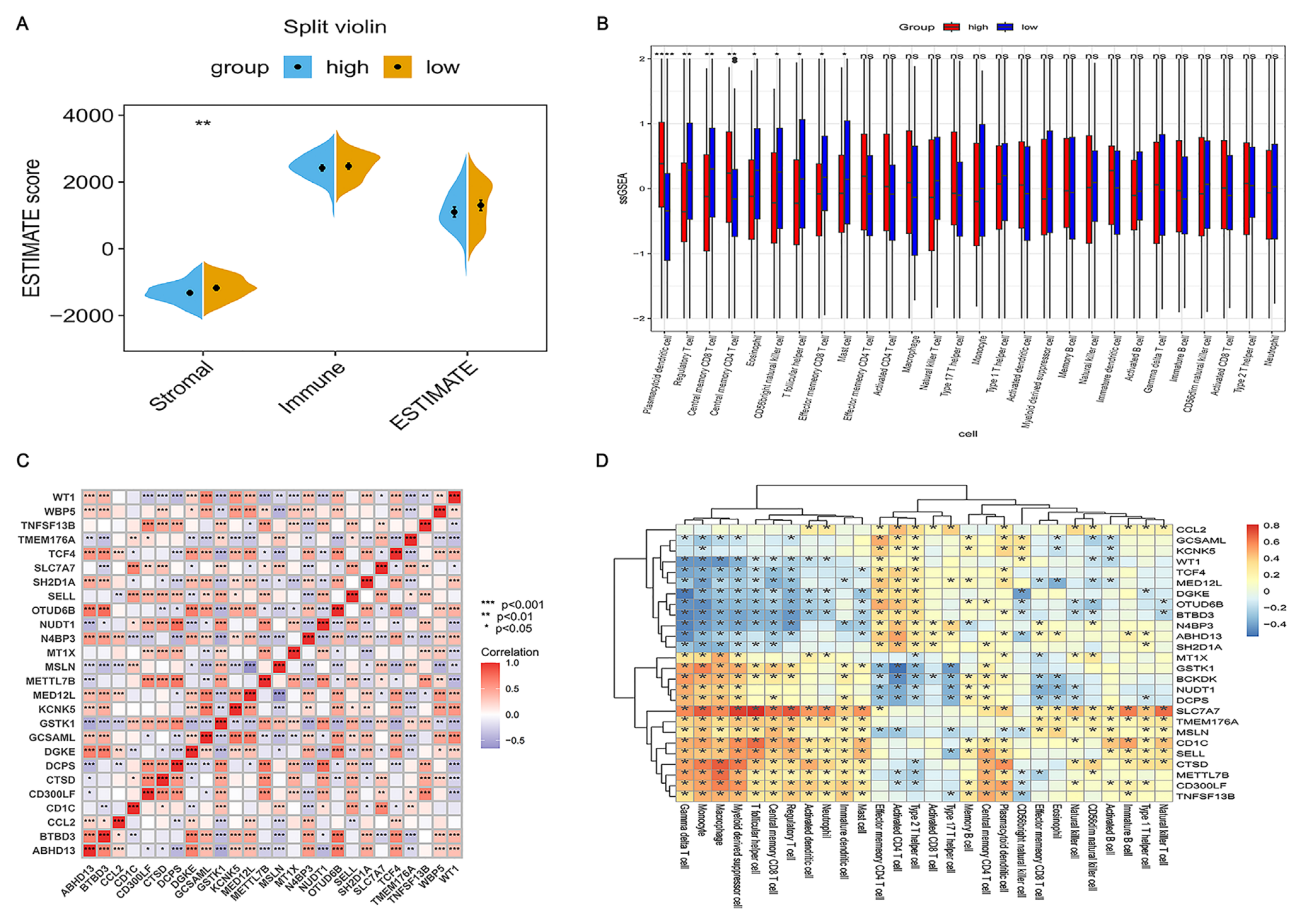
Tumor immune microenvironment and infiltration analysis of immune cells. (A) Stromal score, immune score, and ESTIMATE score in children stratified by high and low risk. (B) ssGSEA‐based analysis of immune cell infiltration in high‐ and low‐risk pediatric patient groups. (C) Heat map of the correlation of 26 prognostic risk model genes. (D) Heat map of the correspondence of 26 prognostic risk model genes with 28 cells with different types of immune infiltration.

To explore the connection between the risk‐scoring model and the bone marrow microenvironment, ESTIMATE algorithm was adopted to figure out the stromal score, immune score, and ESTIMATE score for each patient. As depicted in Figure 6A, a distinct disparity in stromal scores was noticed between the high‐ and low‐risk groups (*p* < 0.01). Subsequently, a detailed analysis of the infiltration degree of 28 types of immune cells was conducted using the ssGSEA algorithm, and the enrichment scores of 28 types of immune cells in the high‐ and low‐risk groups were obtained. The research findings revealed that, among the 28 immune cells, the high‐risk group showed markedly higher infiltration in plasma‐derived dendritic cells and central memory CD4+ T cells than that in the low‐risk group, while the infiltration degree was markedly lower in regulatory T cells (Tregs), central memory CD8+ T cells, eosinophils, CD56 bright natural killer cells, T follicular helper cells (Tfh), effector memory CD8 T cells, and mast cells compared to those in the low‐risk group. This indicated that these genes correlated to RAS may exert an influence on the survival and prognosis of patients with AML by modulating immune‐related pathways.

To further explore the interplay among the 26 survival‐related genes, a heat map was generated. The Spearman correlation coefficient was employed to evaluate the interactions among the identified survival‐related genes. There was a marked association among some genes, as shown in Figure 6C. WT1 was directly proportional to GCSAML and KCNK5, but it was inversely related to DCPS, GSTK1, and TMEM176A. To further explore the interaction of 26 gene signals with immune infiltration in the microenvironment, a heat map was plotted to show the correlation of 26 prognosis‐related genes with 28 immune infiltrating cells. Impressively, SLC7A7 exhibited the most pronounced direct relationship with immune infiltration, specifically with myeloid‐derived suppressor cells and Tfh (Figure 6D). However, DGKE was considerably negatively correlated with immune infiltration, especially in Gamma delta (γδ) T cells as well as CD56 bright natural killer cells.

### 3.6 Prediction of the Sensitivity to Chemotherapy Drugs in Low‐ and High‐Risk Cohorts

**FIGURE 7:**
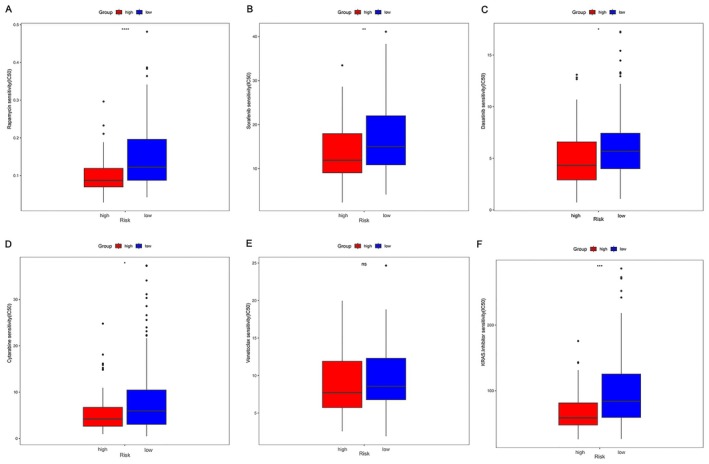
Correlation analysis of risk score and drug sensitivity. (A) Rapamycin; (B) Sorafenib; (C) Dasatinib; (D) Cytarabine; (E) Venetoclax; (F) RAS inhibitors.ns: *p*＞0.05；* *p* ≤ 0.05；** *p* ≤ 0.01；*** *p* ≤ 0.001；**** *p* ≤ 0.001.

Chemotherapy is the most commonly used method for treating AML. A sensitivity analysis was carried out on the risk‐scoring model and therapeutic drugs such as rapamycin, sorafenib, dasatinib, cytarabine, venetoclax, and RAS inhibitors. By comparison with the high‐risk group, the findings revealed a markedly higher drug sensitivity for all drugs in the low‐risk group (Figure 7A–F), except for venetoclax. These results indicate a potentially superior treatment response in the low‐risk group in comparison with the high‐risk group.

### 3.7 Validation of the RNA Expression Levels of Hub Genes

**FIGURE 8:**
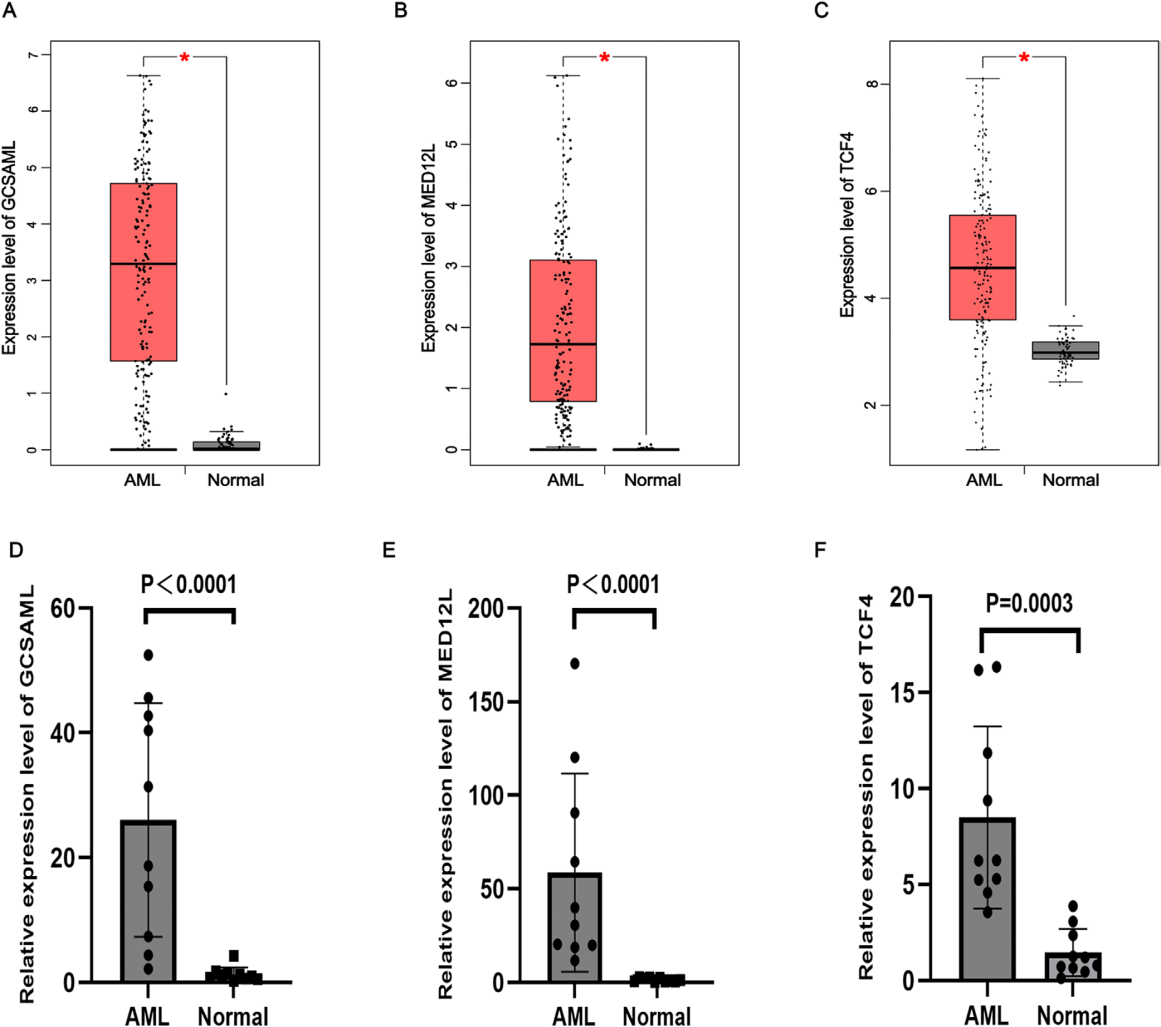
In vitro validation of the RNA expression levels of three genes. The mRNA expression levels of (A) GCSAML, (B) MED12L, (C) TCF4 in normal and AML patients in public databases. The mRNA expression levels of (D) GCSAML (E) MED12L (F) TCF4 in normal and AML patients in clinical samples. * *p* ≤ 0.05.

Previously, the prognostic analysis was performed on both high‐ and low‐risk groups in the training and validation cohorts. Here, three genes (GCSAML, MED12L, TCF4) that had an obvious influence on prognosis (*p* < 0.05, Figures S1 and S2) were selected for validation. First, data from public databases were analyzed through the GEPIA website. The research findings revealed that, for patients with AML, compared with normal individuals, their expression levels of the three genes were markedly elevated (Figure 8A–C). Moreover, 10 newly generated or frozen AML samples (including bone marrow and peripheral blood) were obtained. The expression levels of these three risk genes in primary AML cells were evaluated through qPCR and compared with 10 normal cases. As demonstrated in Figure 8D–F, the expression levels of GCSAML, MED12L, and TCF4 in AML samples were markedly elevated compared to those in normal samples (*p* < 0.05).

## 4 Discussion

AML is a hematopoietic malignancy marked by clonal proliferation of immature myeloid cells, which poses a significant threat to children's lives [13]. The quest to enhance the survival of AML patients remains an ongoing challenge. The RAS oncogene stands out as the most prevalent genetic mutation found in various tumor types. Extensive research has revealed the significant involvement of the RAS signaling pathway in the occurrence and development of hematological malignancies in children [10]. Nevertheless, the clinical relevance of the RAS signaling pathway in pediatric AML remains ambiguous, and further exploration is needed. Within this study, we successfully identified genes associated with the RAS pathway and developed an innovative prognostic risk‐scoring model that enables molecular‐genetic prediction of the survival in AML patients.

First, 236 RAS‐related genes were applied to reveal notable differences in overall survival and immune infiltration between two different RAS gene clusters. The RAS‐B gene cluster was more significant in immune cell infiltration and had a poorer prognosis. Based on the DEGs of these two subtypes, two gene subtypes with different clinical outcomes were identified. Our study findings indicate that RAS signaling pathway‐associated genes might help anticipate the prognosis and response to immunotherapy in AML patients. Thus, we propose a prognostic risk‐scoring model that may assist in informing treatment strategies for AML patients. Finally, a quantitative nomogram was developed by integrating the risk score with relevant clinical characteristics, thereby providing a comprehensive tool that further elucidated the practical utility of the risk scores. The findings of this study denote that AML patients classified in the high‐risk group presented unfavorable prognosis and reduced responsiveness to chemotherapy drugs.

In total, our study encompassed a comprehensive set of 26 genes associated with the RAS signaling pathway. Based on the risk values assigned to each gene, it was found that CD1C, GSTK1, MSLN, SLC7A7, SELL, and TMEM176A were associated with favorable prognosis, while OTUD6B, ABHD13, BTBD3, WT1, METTL7B, NUDT1, DCPS, BCKDK, N4BP3, DGKE, GCSAML, TCF4, KCNK5, SH2D1A, MED12L, CTSD, CD300LF, MT1X, TNFSF13B, and CCL2 were considered as poor prognostic genes in AML patients. Based on the prognostic analysis results of the training and validation sets, we selected three genes (GCSAML, MED12L, TCF4) that had a significant impact on prognosis, so as to verify their expression levels in clinical samples. Both public databases and PCR experiments confirmed notable disparities in the expression levels of GCSAML, MED12L, and TCF4 between normal and AML samples. The germinal center‐associated signaling and motility‐like (GCSAML) proteins, regulated by growth factors and cytokines, are thought to be intricately involved in the proliferation as well as differentiation sites of mature B lymphocytes, exerting a notable influence on the regulation of immune responses and inflammatory processes [14, 15]. After the research, we observed that GCSAML was highly expressed in AML patients, which indicated poor prognosis. This might be related to impaired immune function in AML patients. The protein encoded by MED12L is associated with the transcriptional regulatory mediator complex and participates in the transcriptional coactivator of almost all RNA polymerase II‐dependent genes [16, 17]. Research has shown that MED12L/MED12 is an essential component of the mediator complex and serves as a key regulator of the WNT pathway. Its involvement extends beyond solid tumors, including prostate cancer, where it is implicated in various aspects of tumorigenesis and disease progression [18, 19, 20, 21, 22, 23, 24]. In this study, elevated expression levels of MED12L in AML patients were found to be markedly correlated with an adverse prognosis, which may be related to abnormal activation or inhibition of the WNT signaling pathway by MED12L. Transcription factor 4 (TCF4), a gene encoding a transcription factor, plays a crucial role in regulating gene transcription. An increased expression level of TCF4 has been identified as an independent risk factor associated with unfavorable prognoses among AML patients [25]. In our survival analysis, TCF4 also indicated poor prognosis.

Our study showed that compared to traditional AML risk stratification, the risk score generated in our analysis demonstrated enhanced predictive capabilities in determining the prognosis of patients with AML. By utilizing our risk score, AML patients were effectively stratified into high‐ and low‐risk groups. By comparison with the low‐risk group, survival analysis revealed observably poorer prognoses in the high‐risk group. ROC analysis revealed that the AUC values for the 1‐, 3‐, and 5‐year survival of AML patients exceeded 0.8, indicating a high level of accuracy in predicting patient outcomes. The uniCox as well as multiCox regression analyses consistently illustrated that the risk score was an independent prognostic factor. Combining traditional AML risk stratification analysis, we noticed that the high‐risk group exhibited elevated risk scores, indicating a greater likelihood of adverse outcomes. This conclusion conformed to clinical observation and further confirmed that our risk‐scoring model served as an effective tool for prognostic guidance. Notably, we developed a prognostic nomogram that integrated both risk scores and clinical characteristics, thereby enhancing the practical applicability of our risk‐scoring model in clinical practice.

Notoriously, the tumor microenvironment encompasses a complex ecosystem comprising diverse immune cells, which collectively exert a pivotal influence on the tumor occurrence, progression, and therapeutic outcomes [26]. The infiltration of tumor microenvironment is of considerable significance for the pathogenesis and progression of AML [27, 28]. Early studies have suggested that immune evasion is an important factor for the progression or recurrence of AML [29, 30]. Therefore, immunotherapy provides a new therapeutic option for AML patients [31]. We conducted an analysis to delve into the connection of the risk‐scoring model with immune infiltration in 28 cells. Our findings revealed a notable correlation in the high‐risk group and enhanced infiltration of plasmacytoid dendritic cells, resulting in a poor prognosis. These findings align with the results revealed by Zhu et al. [32], and such patients might develop resistance to drugs such as cytarabine. Further research was conducted on the correlation of 26 genes with immune infiltrating cells, and it was found that SLC7A7 showed a positive correlation with immune infiltrating cells. SLC7A7 encodes a carrier of transport proteins called lysinuric proteins, which are associated with the transport of lysine, arginine, and other proteins [33]. The metabolism of arginine is intricately linked to the activation of macrophages and T cells [34]. Therefore, SLC7A7 can modify the function of immune cells, including macrophages and T cells, through the transport of arginine. In our study, elevated expression of SLC7A7 was found to be correlated with heightened infiltration of various immune cell populations, involving myeloid‐derived suppressor cells, macrophages, monocytes, Tfh, and Tregs, which was consistent with previous research findings [35, 36]. A substantial amount of evidence indicates that RAS genes play a functional role in the recruitment, activation, and differentiation of immune cells, as well as in coordinating tumor cells to evade immune surveillance [11, 12]. Therapies targeting RAS genes and related signaling pathways are highly effective for treating tumors with RAS‐activating mutations. Existing studies have shown that treatment methods targeting RAS pathways have achieved notable effects in solid tumors such as non‐small cell lung cancer, colorectal cancer, and prostate cancer [37, 38, 39, 40, 41, 42, 43, 44]. In our risk scoring model, constructed based on RAS genes, significant differences in the immune microenvironment are observed among different risk groups, which may provide insights into the application of targeted therapy for RAS pathways in pediatric AML.

Chemotherapy is the principal treatment option for AML, and the recurrence of AML following chemotherapy represents a notable factor impacting the long‐term survival of patients. Poor therapeutic effect or recurrence may be related to drug resistance in tumors [45]. Therefore, we conducted an investigation to assess the predictive capability of risk scoring in determining chemotherapy response. Our findings revealed notable differences in drug sensitivity within the low‐ and high‐risk groups across the six drugs (rapamycin, sorafenib, dasatinib, cytarabine, venetoclax, RAS inhibitors). Specifically, the low‐risk group demonstrated evidently higher drug sensitivity for all drugs except venetoclax. These findings pointed out that AML patients in the high‐risk group presented relatively inferior chemotherapy response in comparison with those in the low‐risk group. This could be one of the causes of the unfavorable prognosis observed in the high‐risk group. The results obtained suggest that the risk‐scoring model we developed has a certain degree of predictive capability in determining chemotherapy response.

However, certain limitations are present in our research. First, this study was conducted based on retrospective data, and the limited sample sizes of the training set and validation set may affect the statistical significance and generalizability of the results. Validation of the predictive capacity of the risk‐scoring model and nomogram warrants additional investigation through multicenter or prospective studies. Second, the roles of these 26 genes in AML necessitate additional in vitro and in vivo investigations. Furthermore, the mechanisms of these genes in the occurrence, progression, immunotherapy, and drug sensitivity of AML also require further exploration, offering innovative strategies for the treatment of AML.

Briefly, we have generated a prognostic risk‐scoring model based upon RAS pathway genes and confirmed that this model demonstrated promising effectiveness in predicting the outcomes of AML patients, revealing distinct variations in clinical characteristics, tumor microenvironment infiltration, drug sensitivity, and risk scores. Therefore, this model offers novel perspectives that can substantially contribute to the prognosis and treatment of patients with AML. Combining our model with traditional comprehensive treatment strategies may help further improve the overall survival rate of pediatric AML.

## Author Contributions

All authors contributed to the study conception and design. Writing – original draft preparation: Cai‐Ju Luo. Writing – review and editing: Cai‐Ju Luo and Lu‐Hong Xu. Conceptualization: Cai‐Ju Luo. Methodology: Cai‐Ju Luo. Formal analysis and investigation: Cai‐Ju Luo and Ming‐Liang Rao. Funding acquisition: Jian‐Pei Fang and Lu‐Hong Xu. Resources: Yilimuguli·abudukeremu. Supervision: Dun‐Hua Zhou and Yang Li. All authors commented on previous versions of the manuscript, and read and approved the final manuscript.

## Ethics Statement

This study was granted ethical approval from the Ethics Committee of SUN Yat‐Sen Memorial Hospital, SUN Yat‐Sen University.

## Consent

All informed consent was obtained from the subject(s) and/or guardian(s).

## Conflicts of Interest

The authors declare no conflicts of interest.

## Supporting information


**Figure S1.** Survival analysis of 26 risk genes in the training cohort. (A) ABHD13; (B) BCKDK; (C) BTBD3; (D) CCL2; (E) CD1C; (F) CD300LF; (G) CTSD; (H) DCPS; (I) DGKE; (J) GCSAML; (K) GSTK1; (L) KCNK5; (M) MED12L; (N) METTL7B; (O) MSLN; (P) MT1X; (Q) N4BP3; (R) NUDT1; (S) OTUD6B; (T) SELL; (U) SH2D1A; (V) SLC7A7; (W) TCF4; (X) TMEM176A; (Y) TNFSF13B; (Z) WT1.

**Figure S2.** Survival analysis of 26 risk genes in the validation cohort. (A) ABHD13; (B) BCKDK; (C) BTBD3; (D) CCL2; (E) CD1C; (F) CD300LF; (G) CTSD; (H) DCPS; (I) DGKE; (J) GCSAML; (K) GSTK1; (L) KCNK5; (M) MED12L; (N) METTL7B; (O) MSLN; (P) MT1X; (Q) N4BP3; (R) NUDT1; (S) OTUD6B; (T) SELL; (U) SH2D1A; (V) SLC7A7; (W) TCF4; (X) TMEM176A; (Y) TNFSF13B; (Z) WT1.

## Data Availability

All data generated or analyzed during this study are included in this published article and its Supporting Information files.
