# Supplementary figures and images for: A Novel Prognostic Risk‐Scoring Model Based on RAS Gene‐Associated Cluster in Pediatric Acute Myeloid Leukemia
Source: Cancer Med. 2025 Mar 10;14(5):e70716. doi: 10.1002/cam4.70716 (PMC11891924; doi:10.1002/cam4.70716)

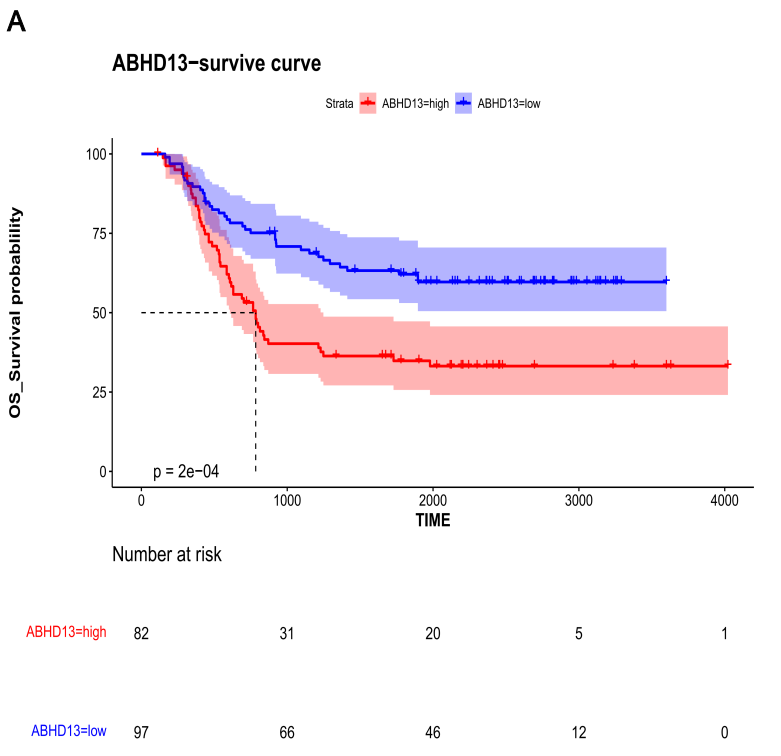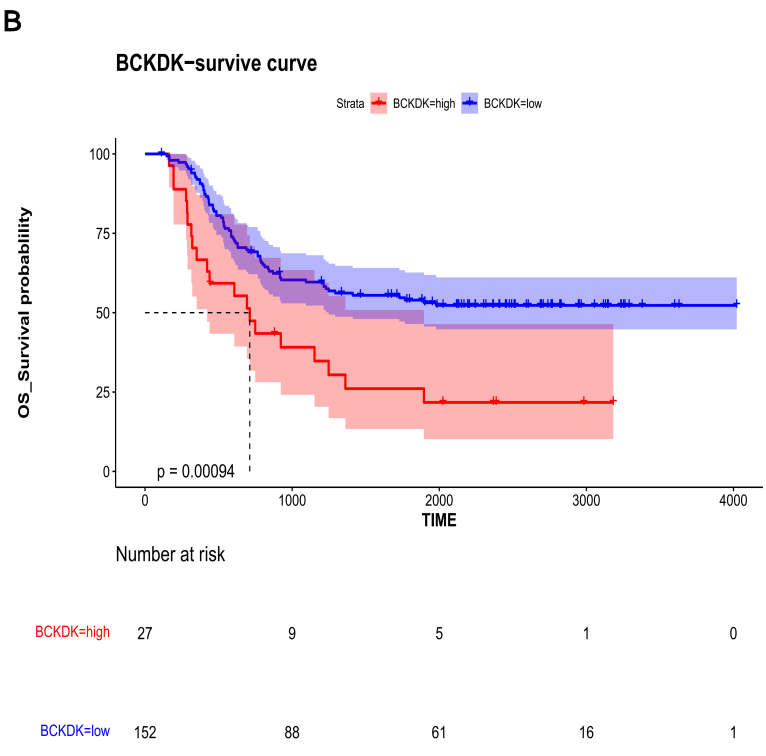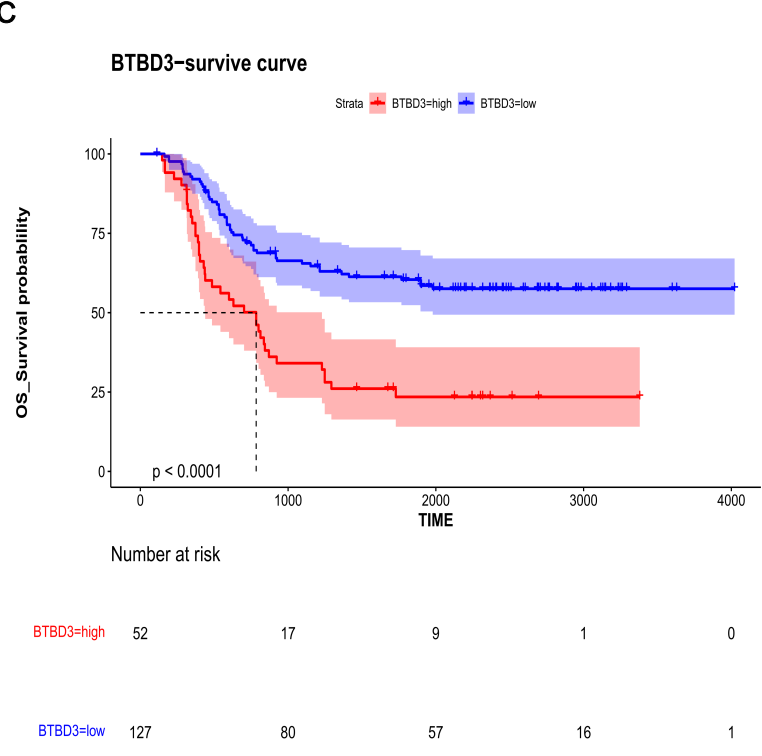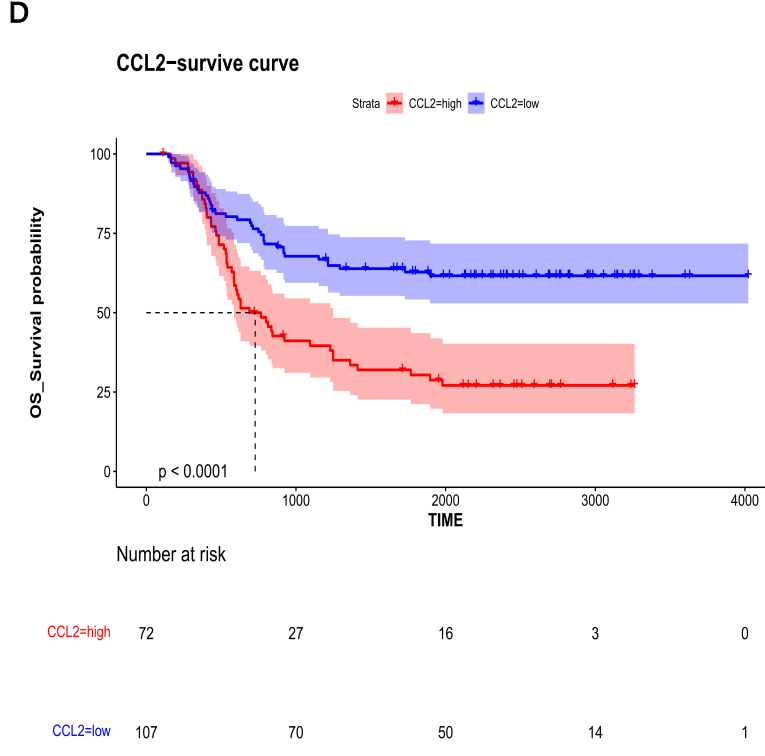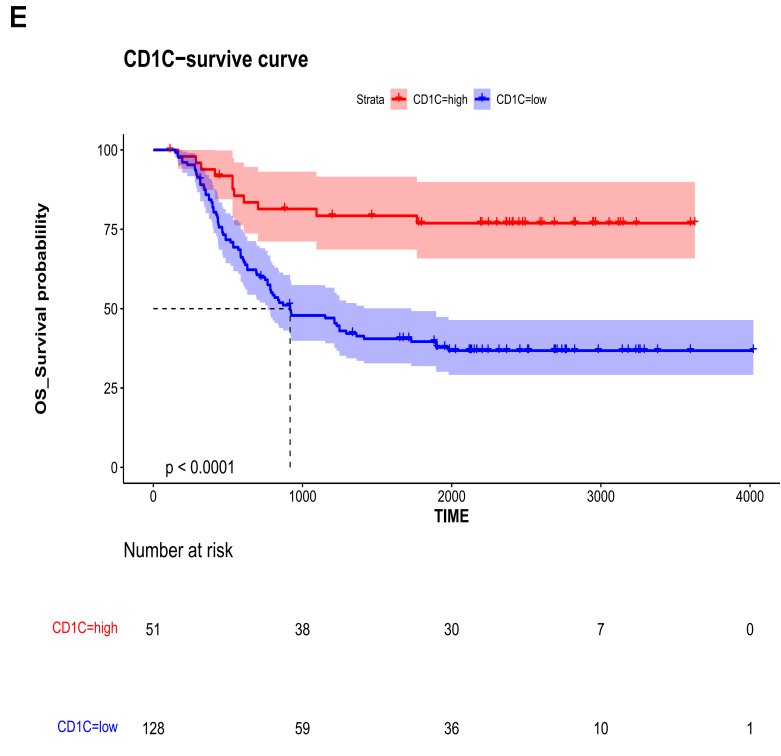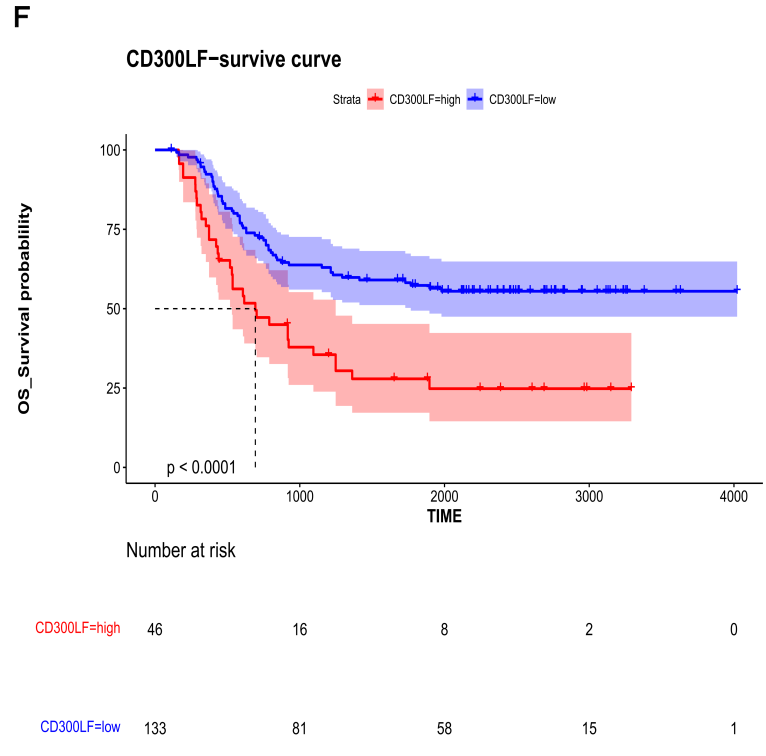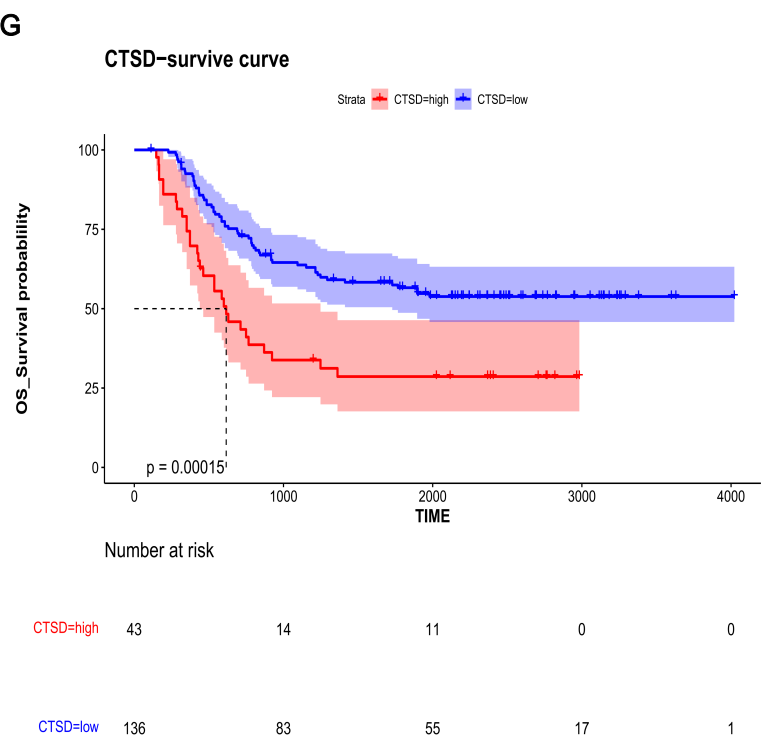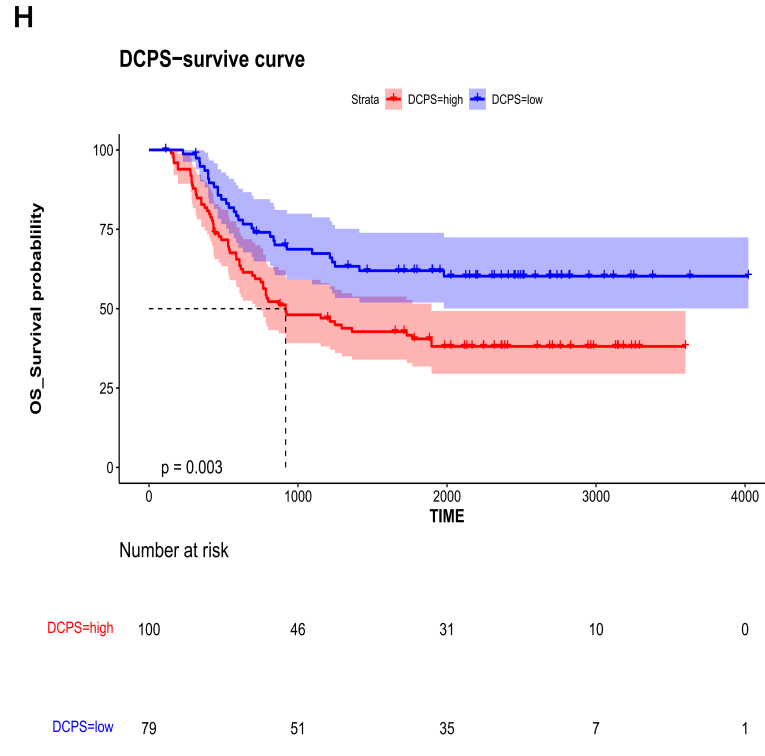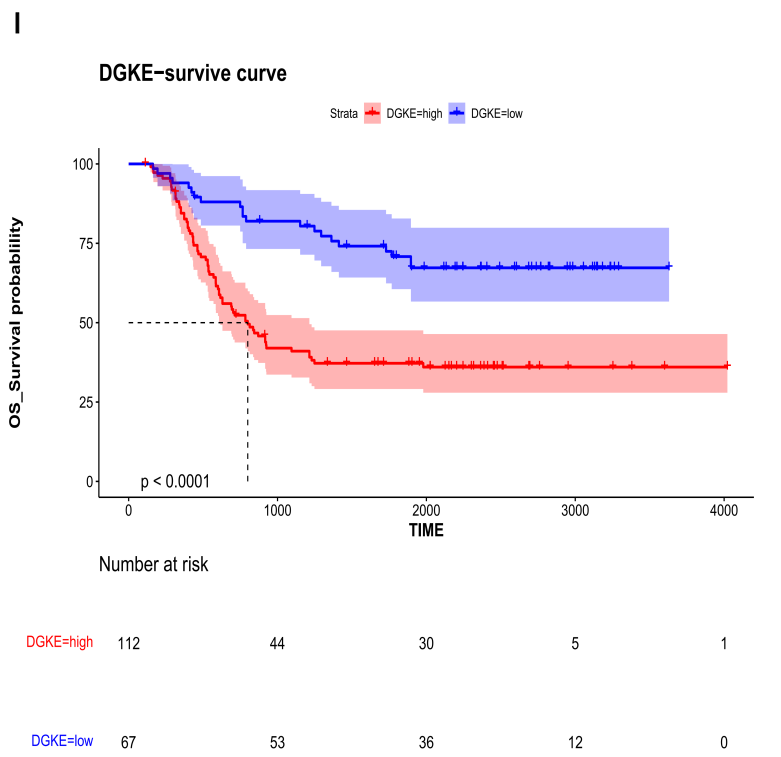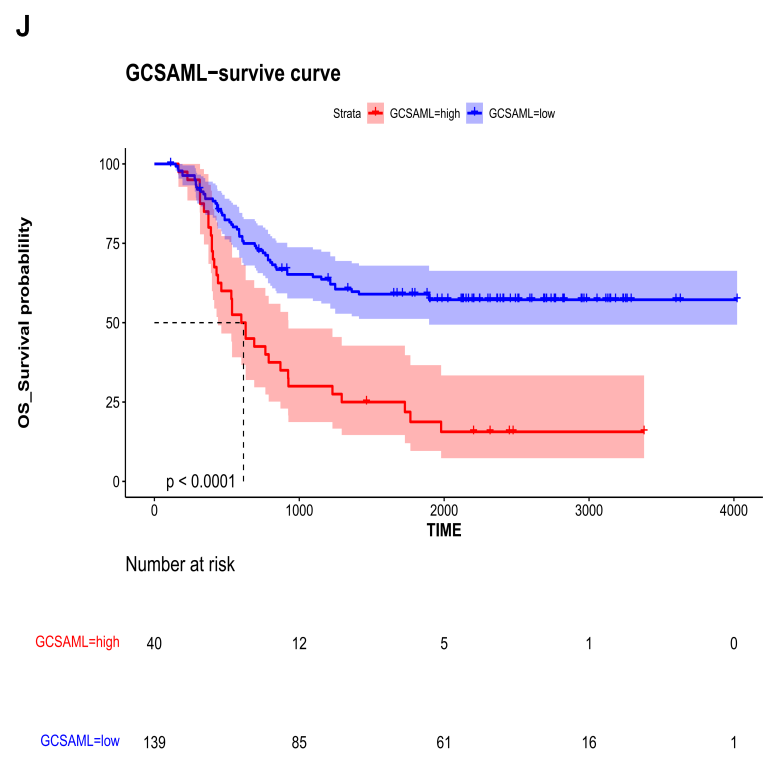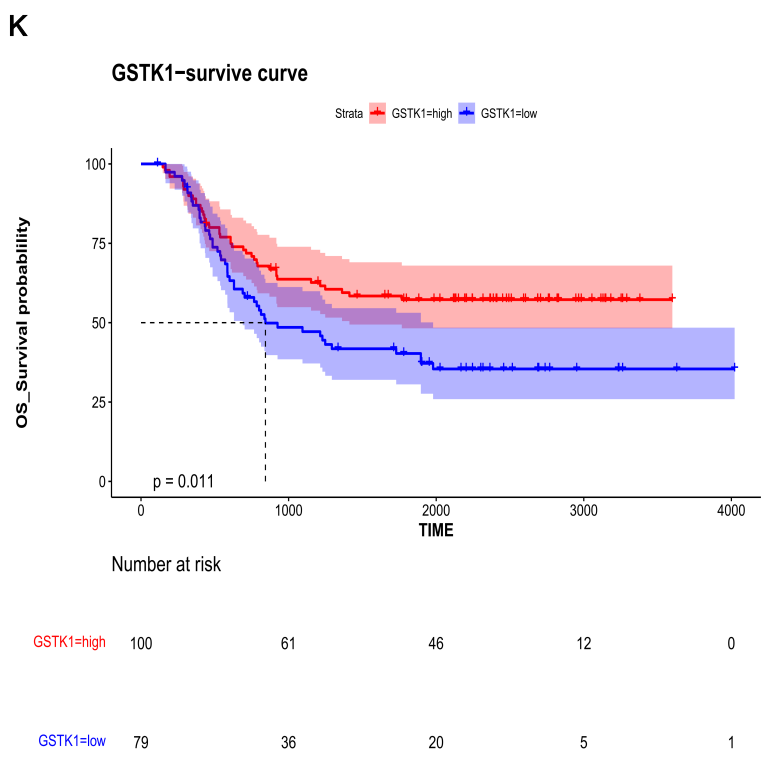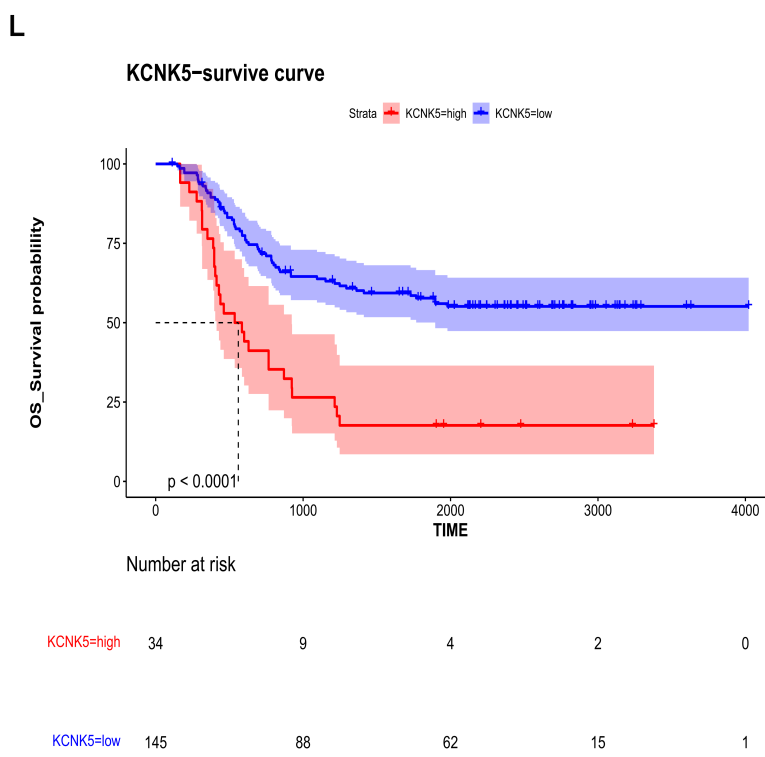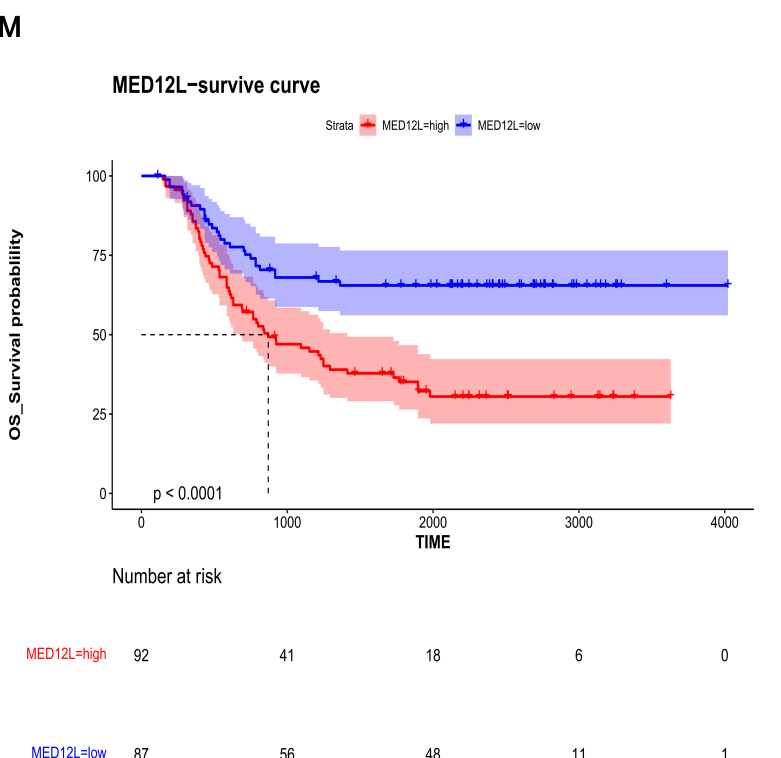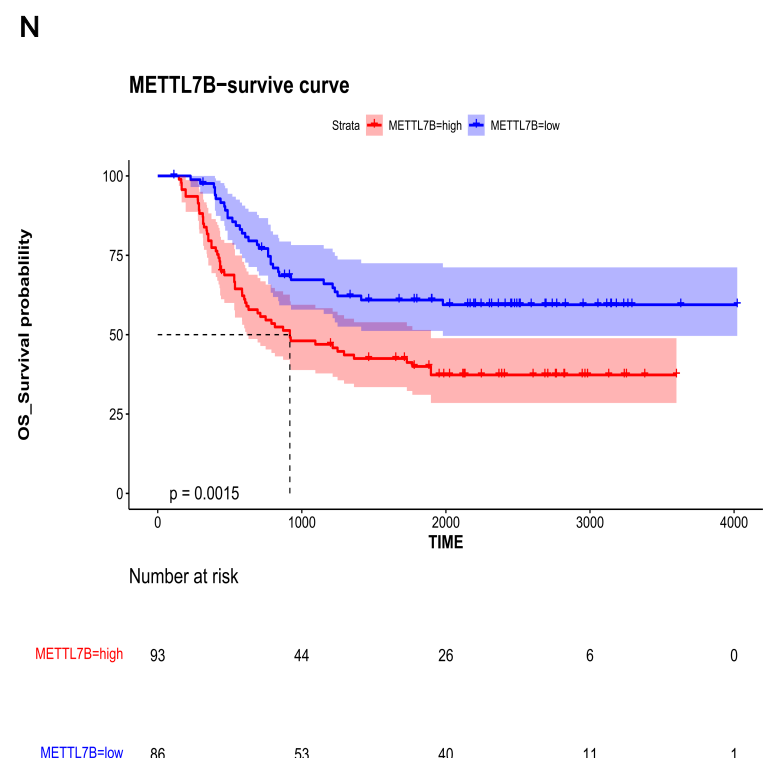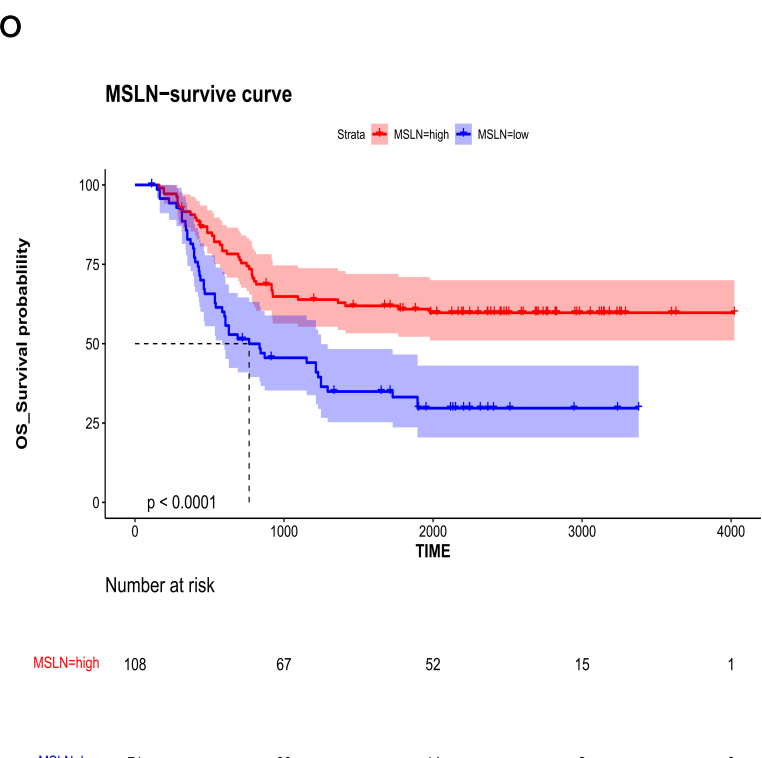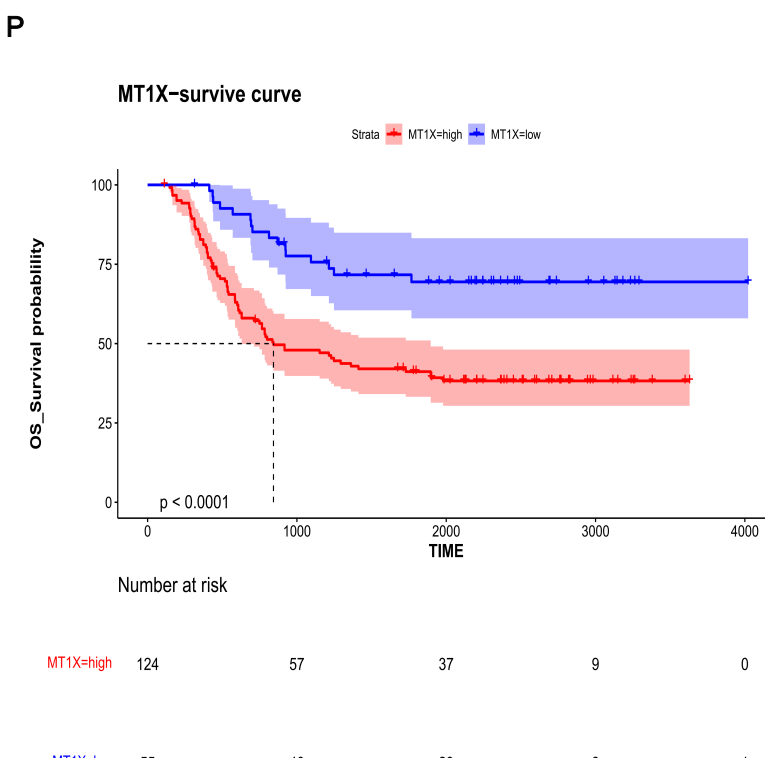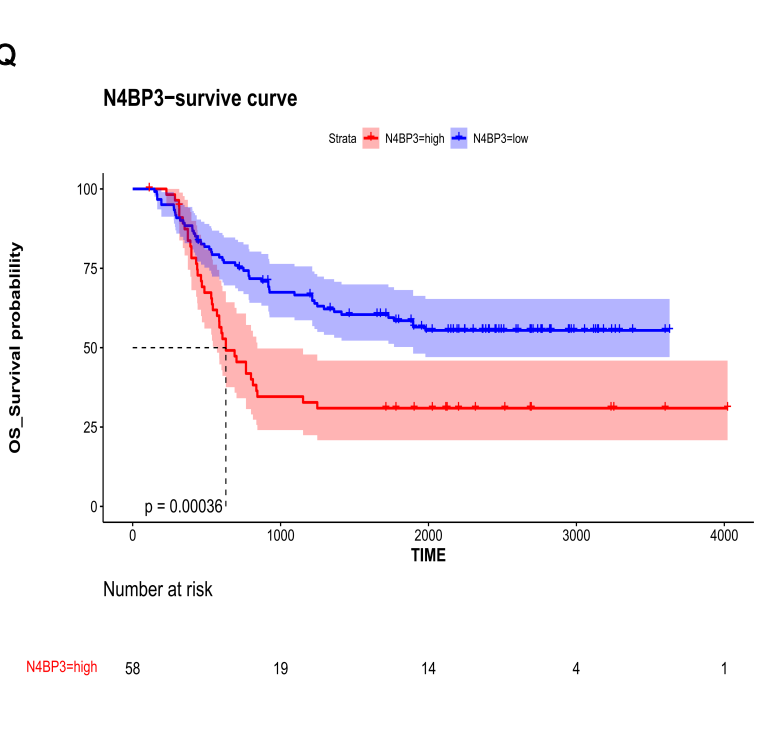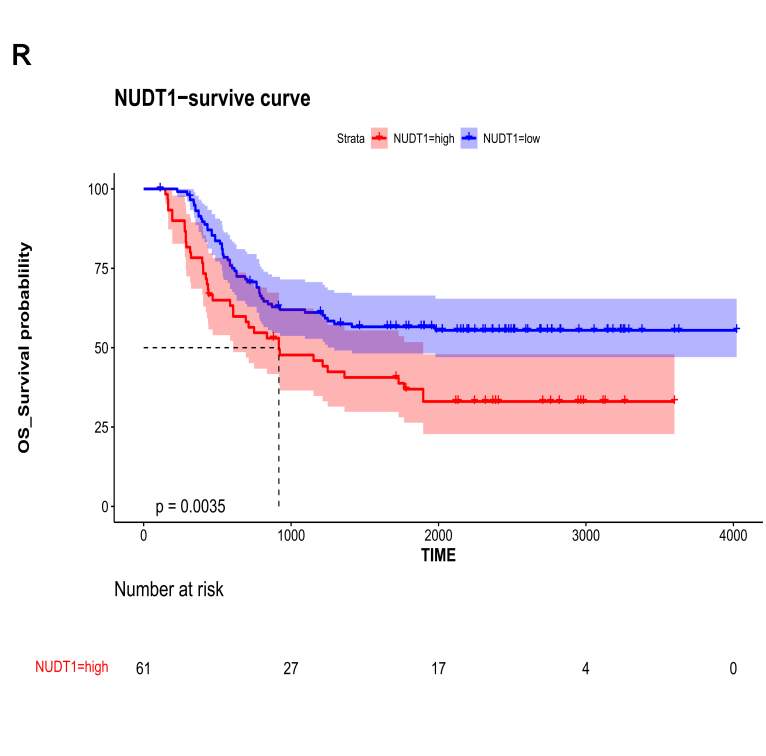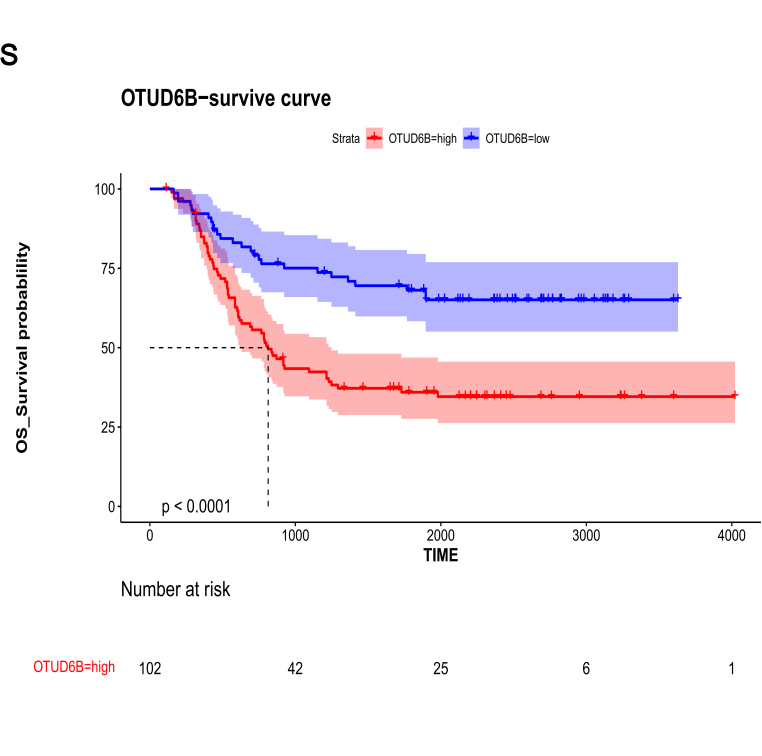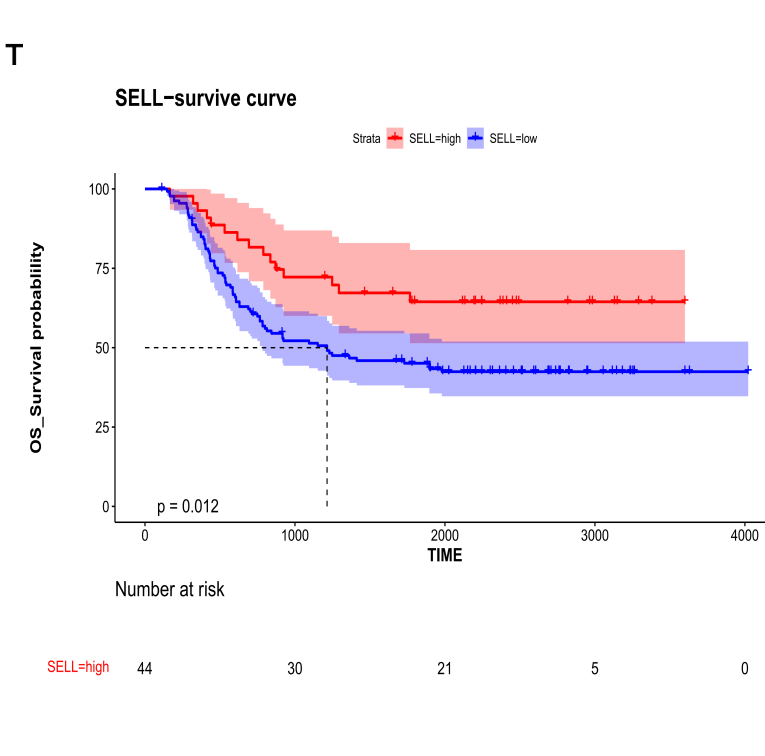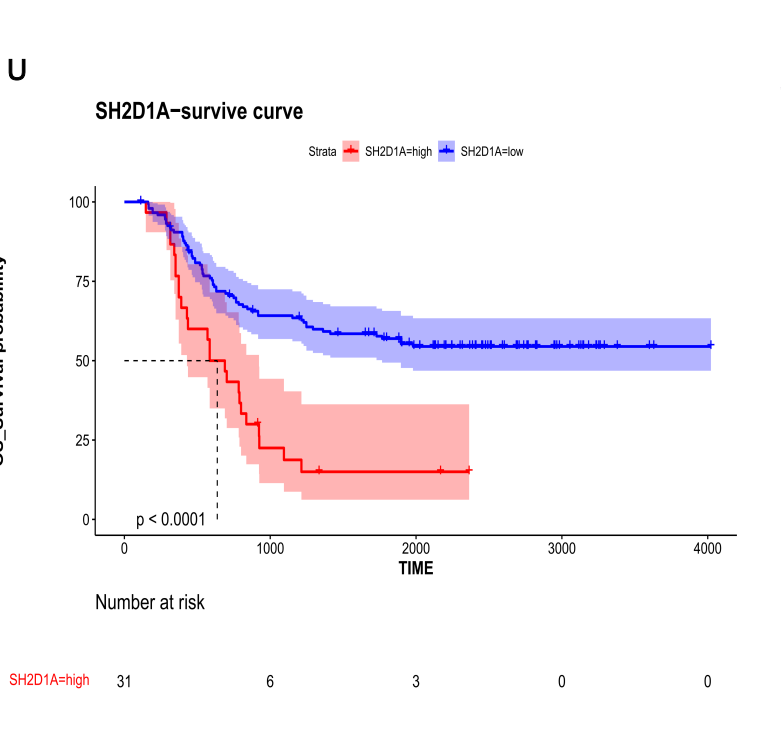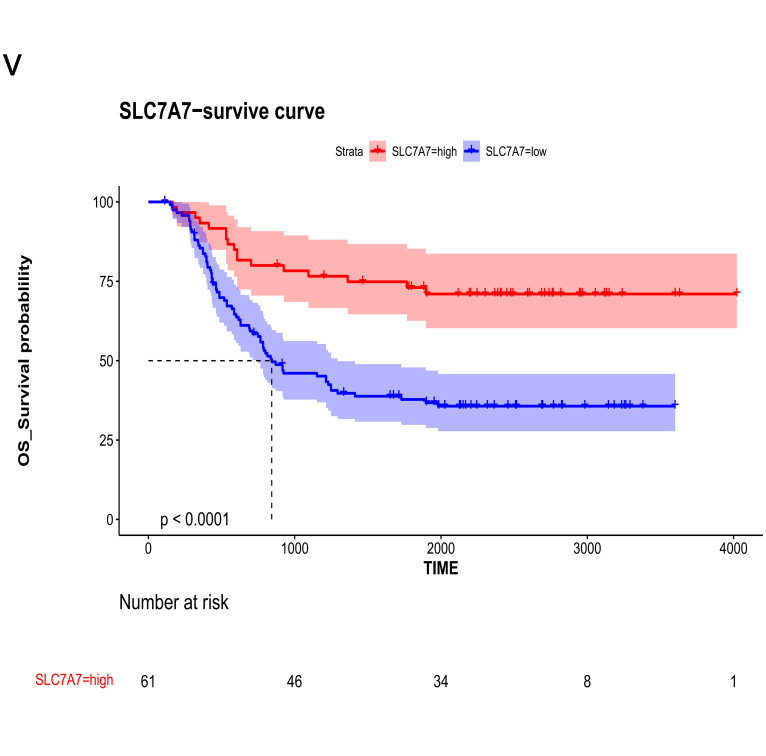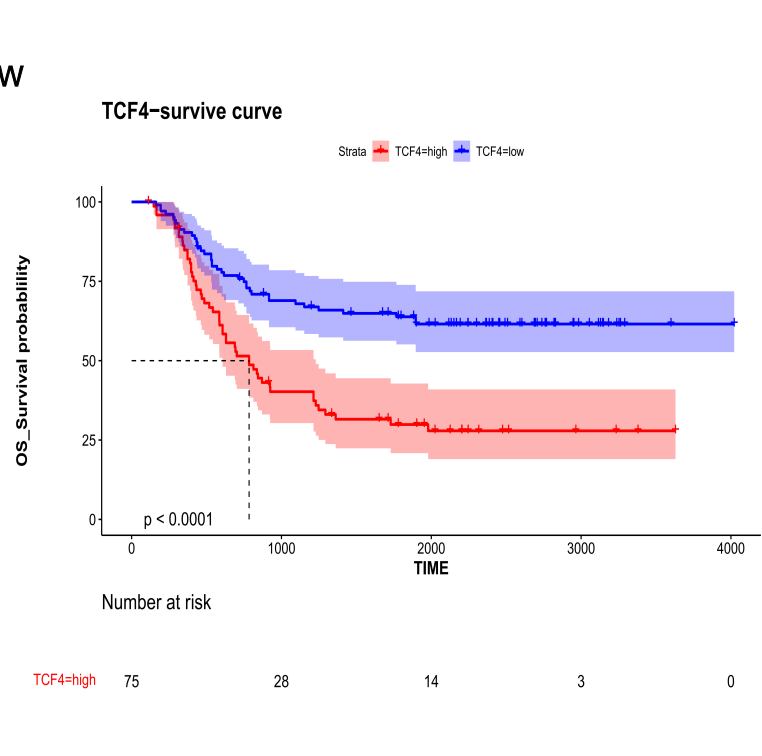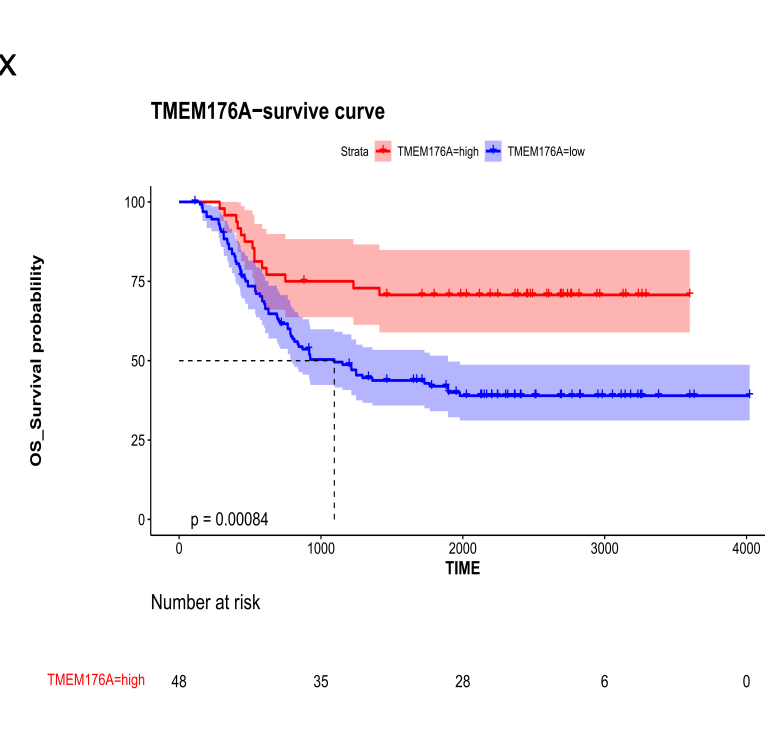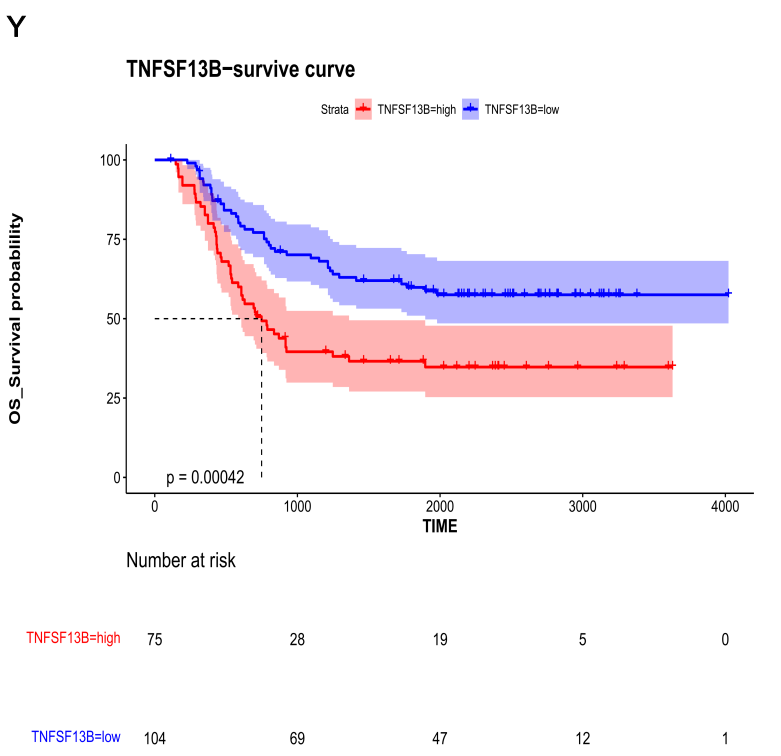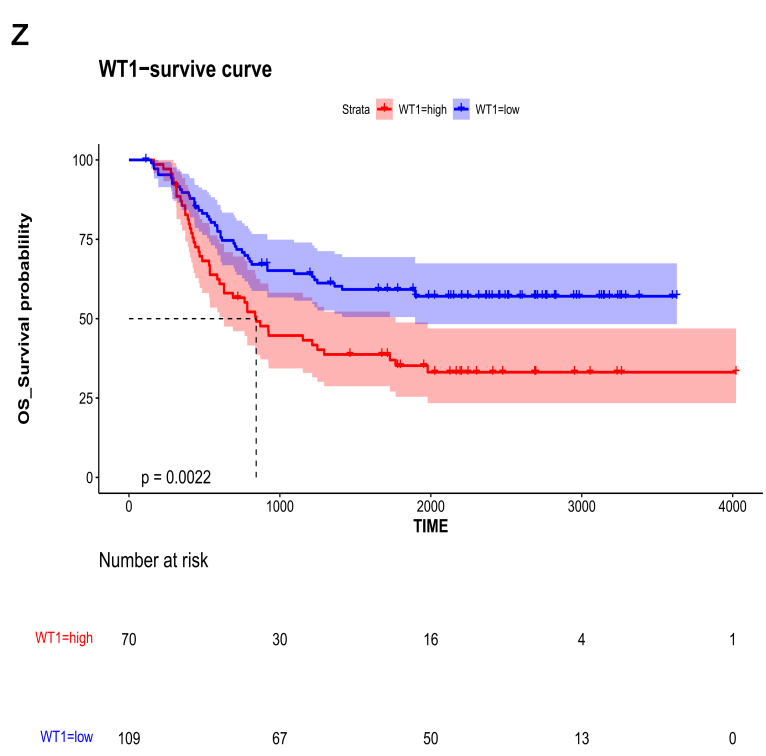

Supplement: Supplementary file 1 — Figure S1. Survival analysis of 26 risk genes in the training cohort. (A) ABHD13; (B) BCKDK; (C) BTBD3; (D) CCL2; (E) CD1C; (F) CD300LF; (G) CTSD; (H) DCPS; (I) DGKE; (J) GCSAML; (K) GSTK1; (L) KCNK5; (M) MED12L; (N) METTL7B; (O) MSLN; (P) MT1X; (Q) N4BP3; (R) NUDT1; (S) OTUD6B; (T) SELL; (U) SH2D1A; (V) SLC7A7; (W) TCF4; (X) TMEM176A; (Y) TNFSF13B; (Z) WT1. [file CAM4-14-e70716-s001.pdf]

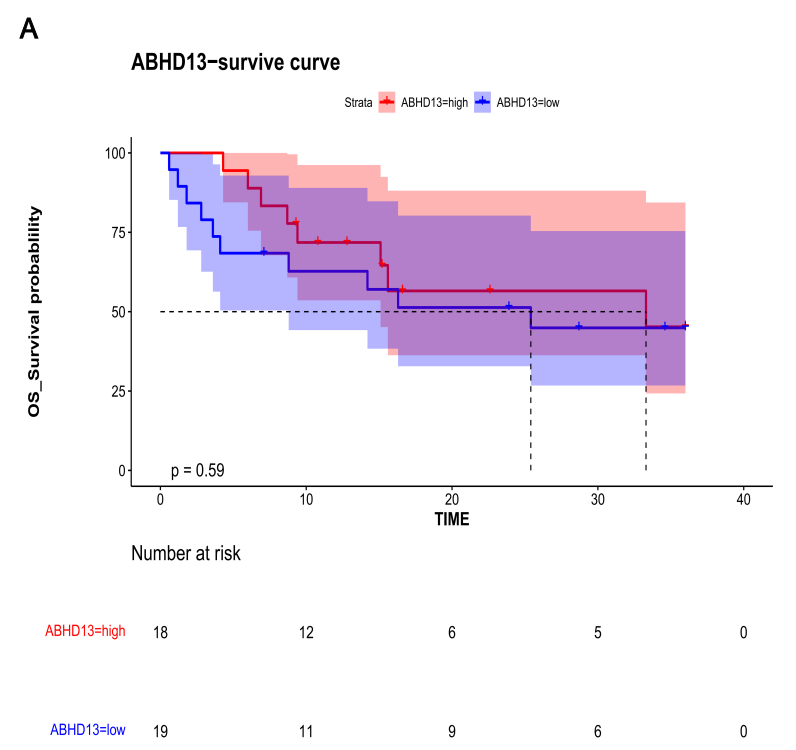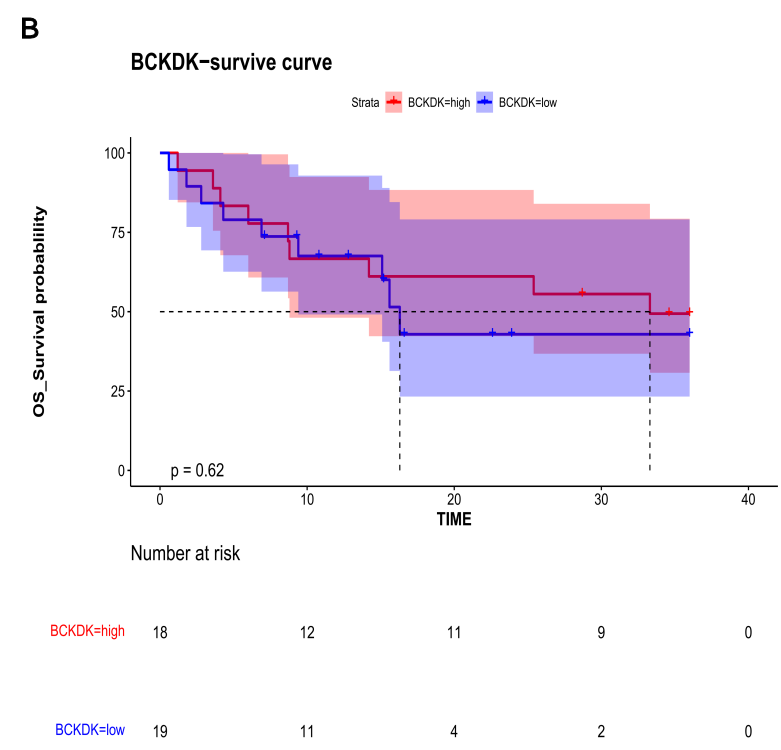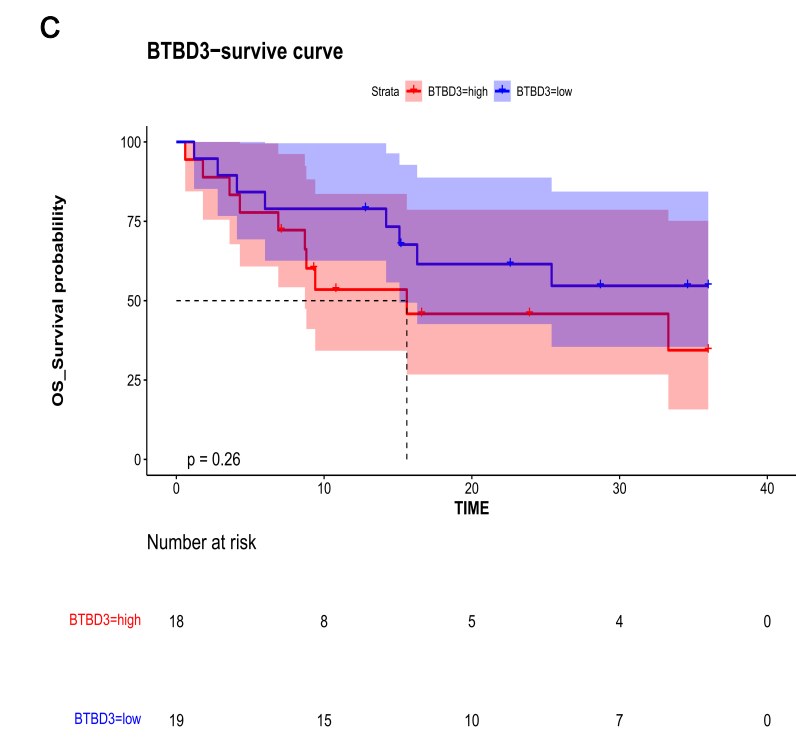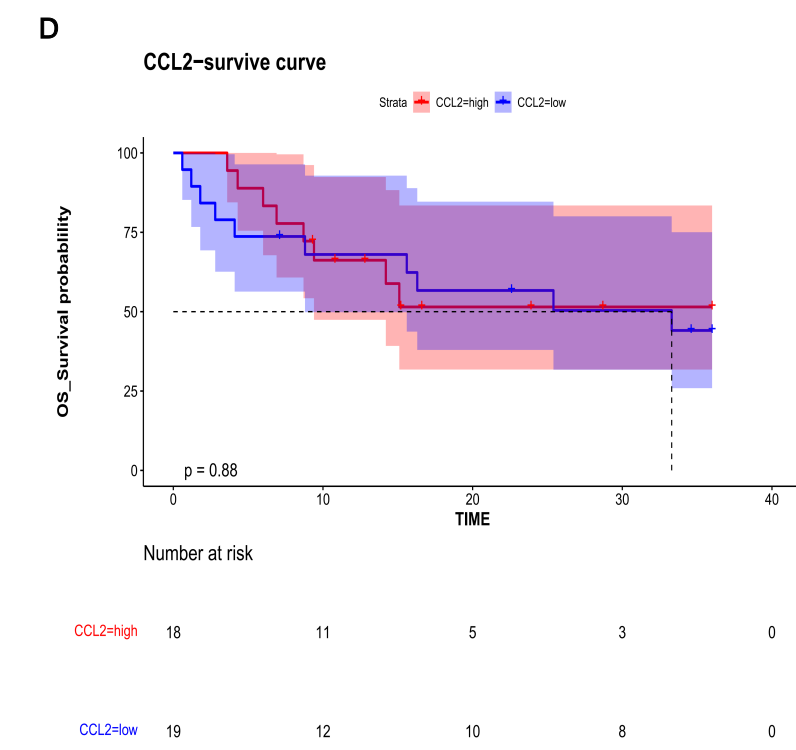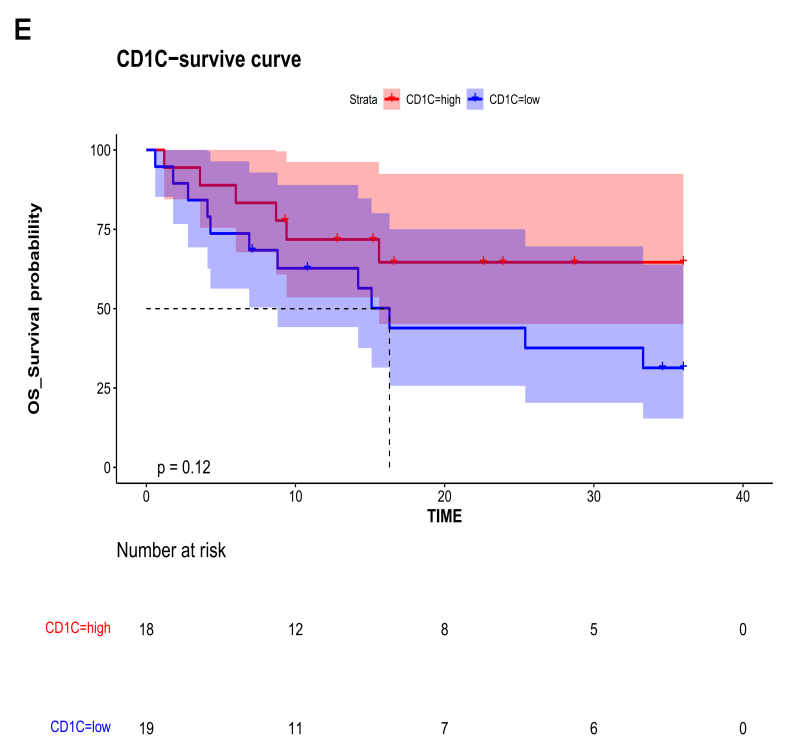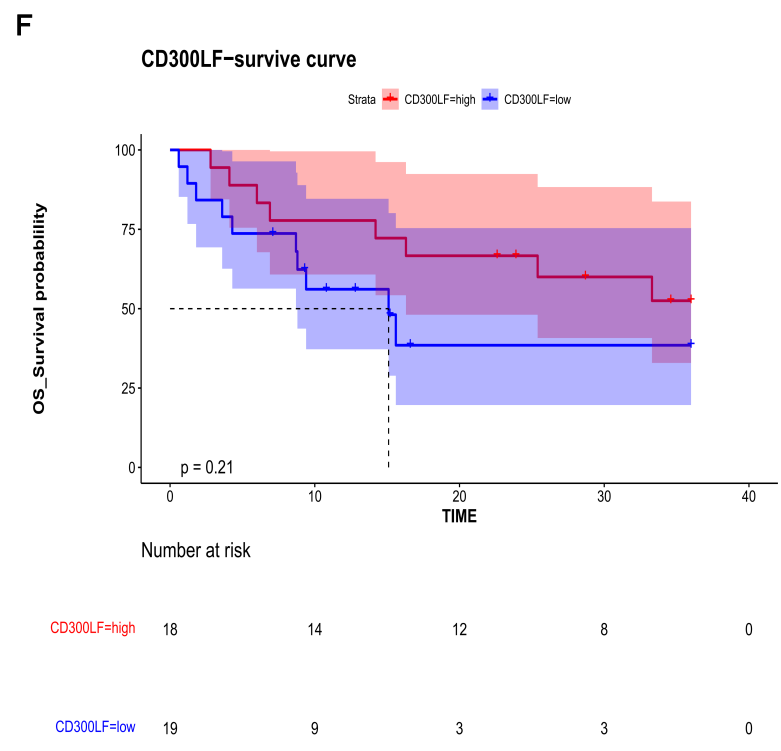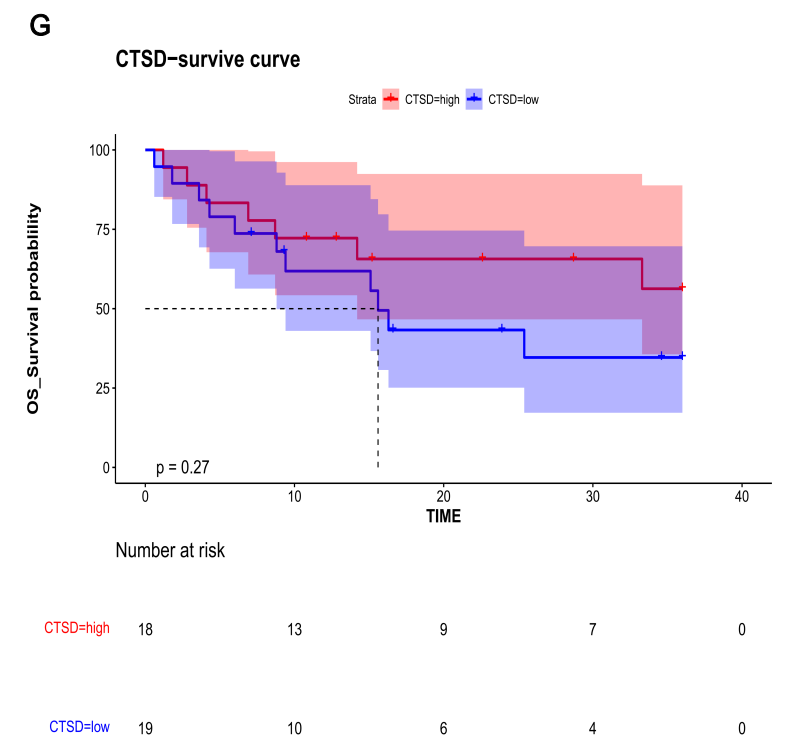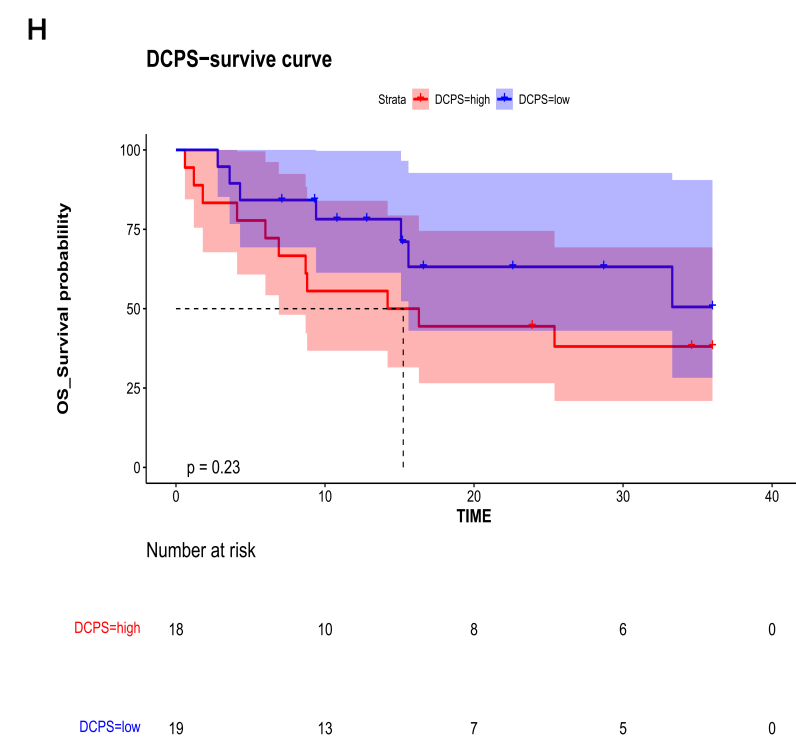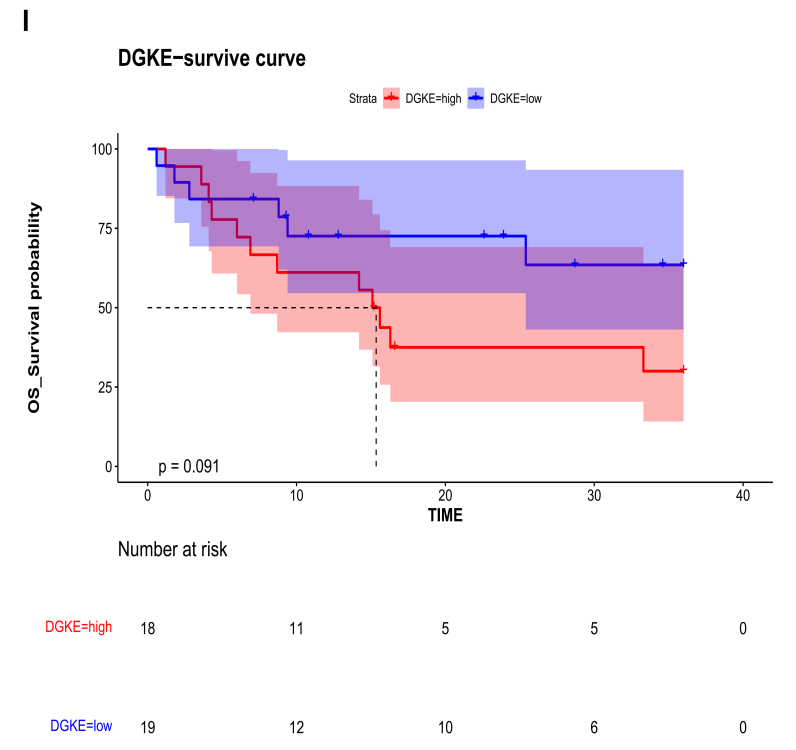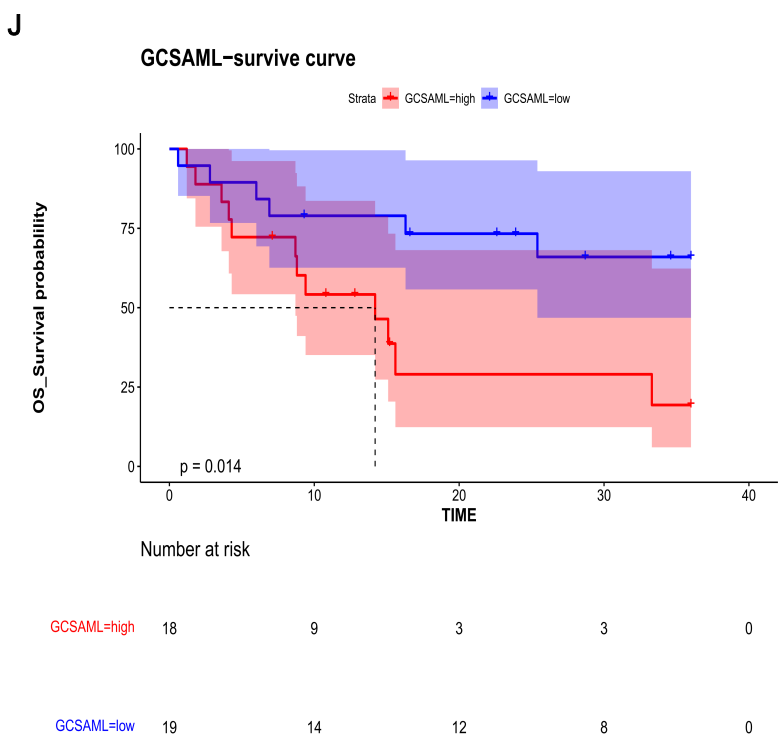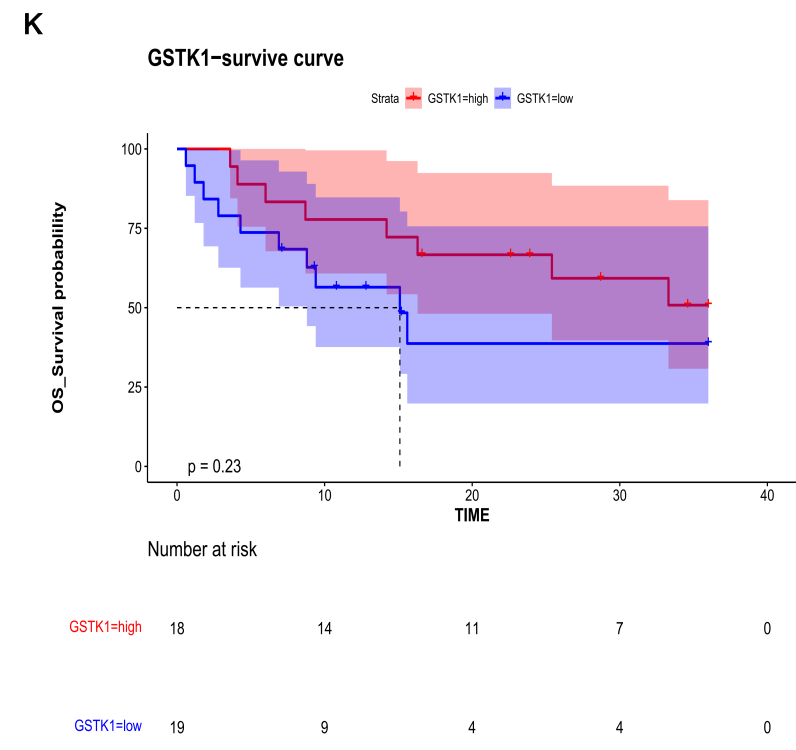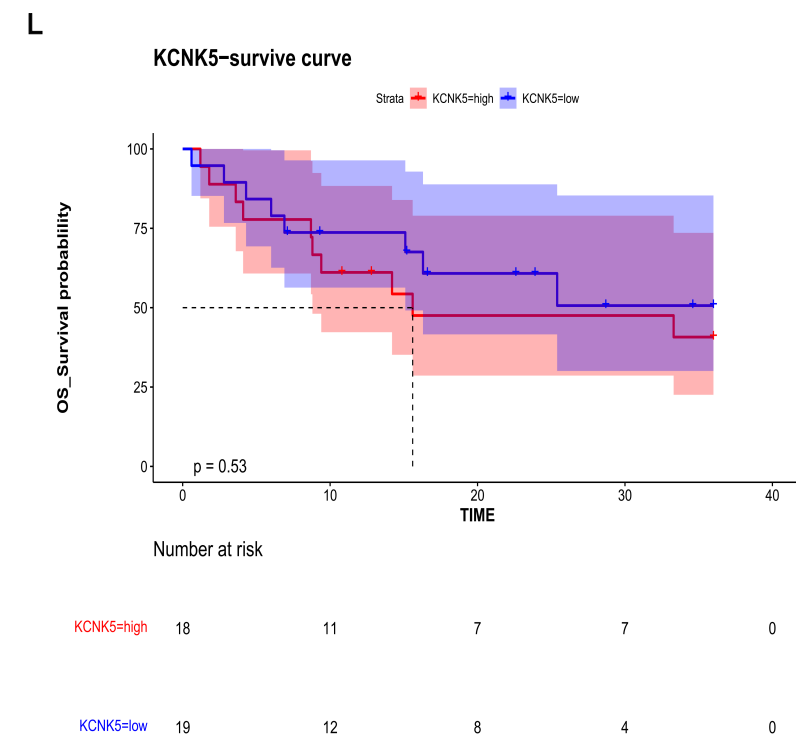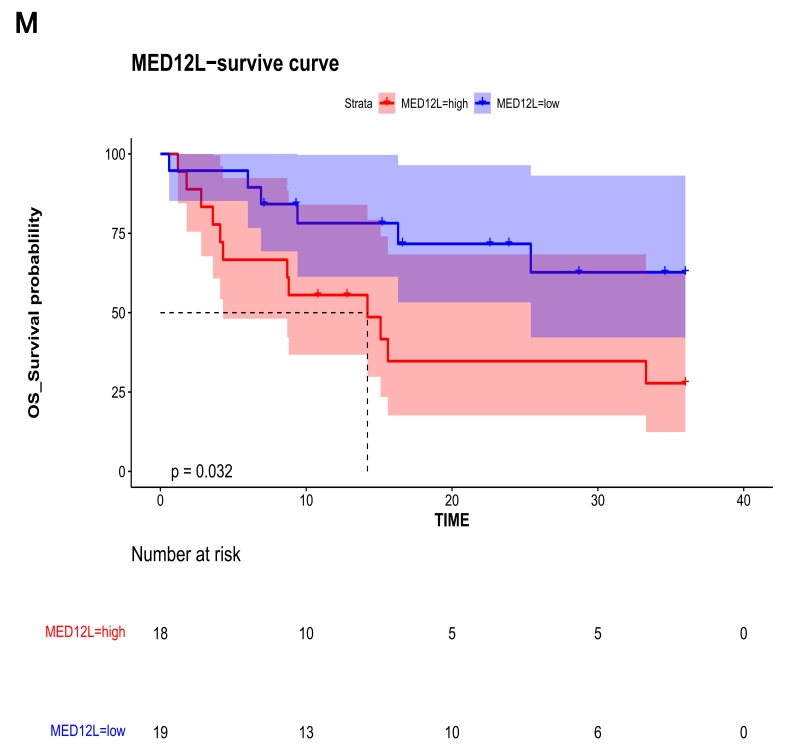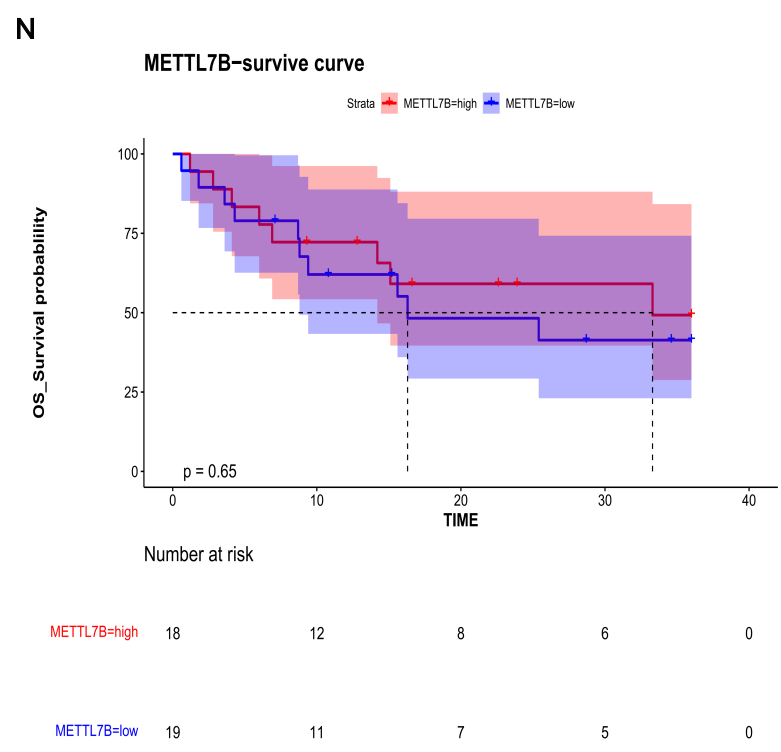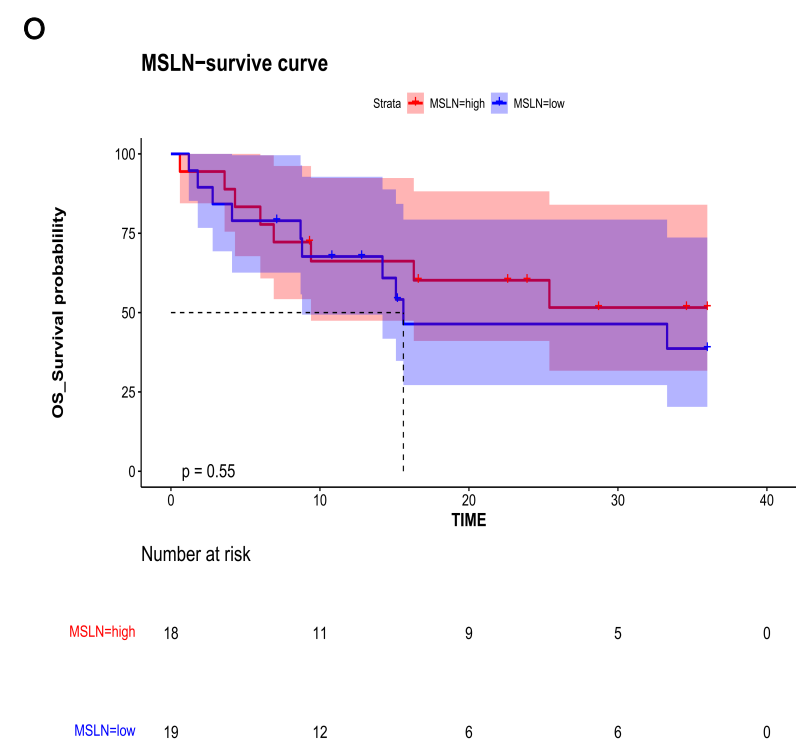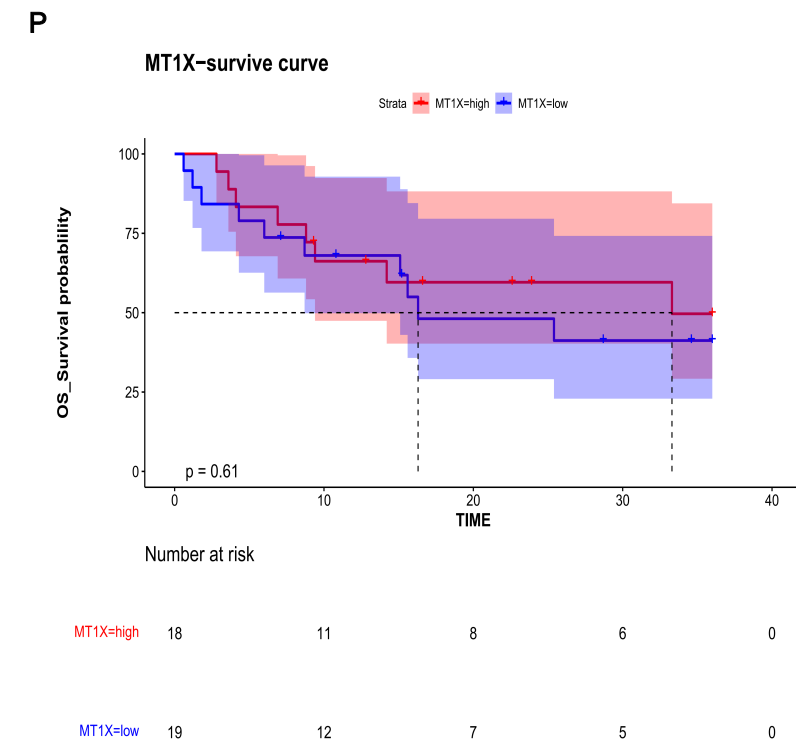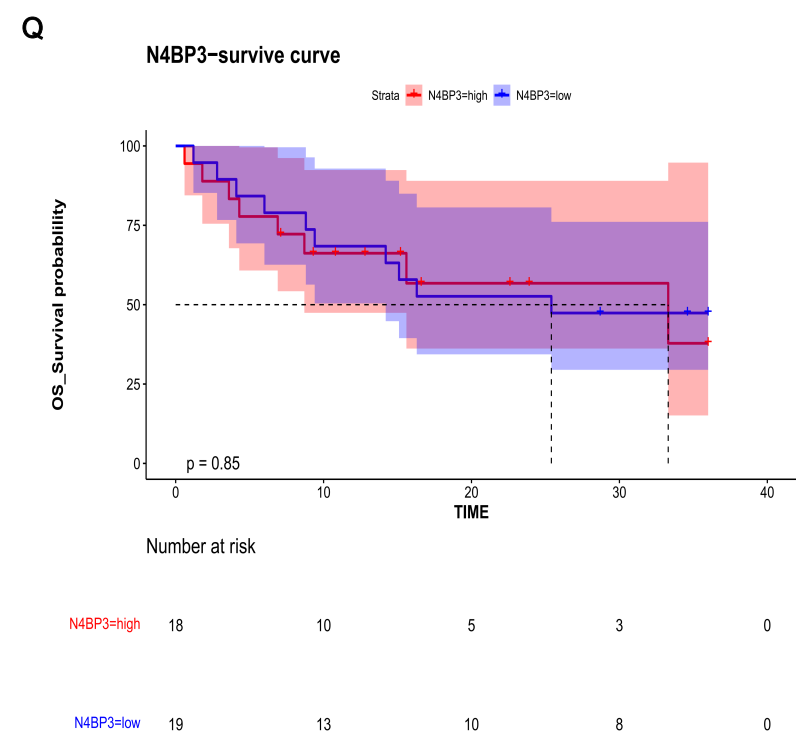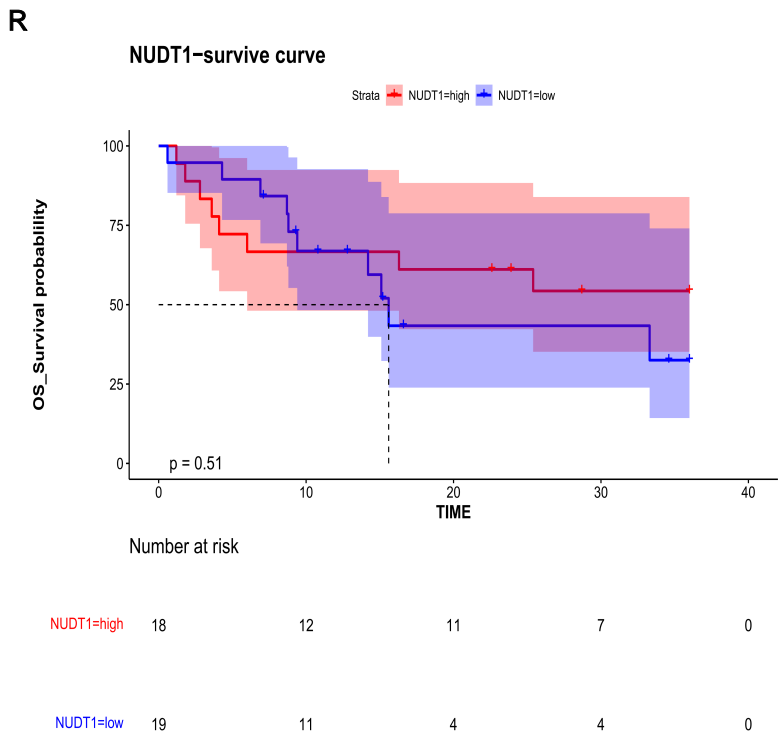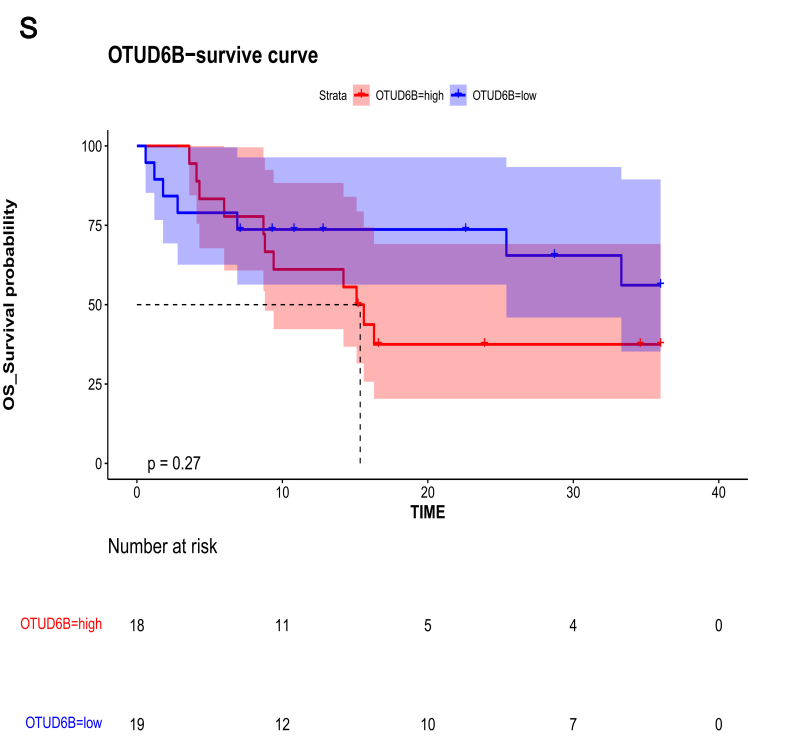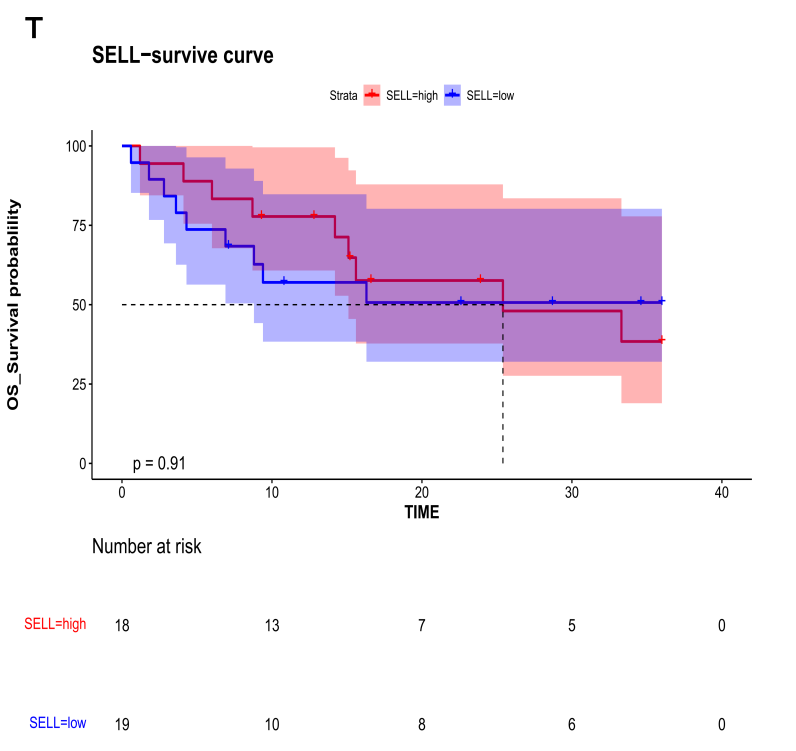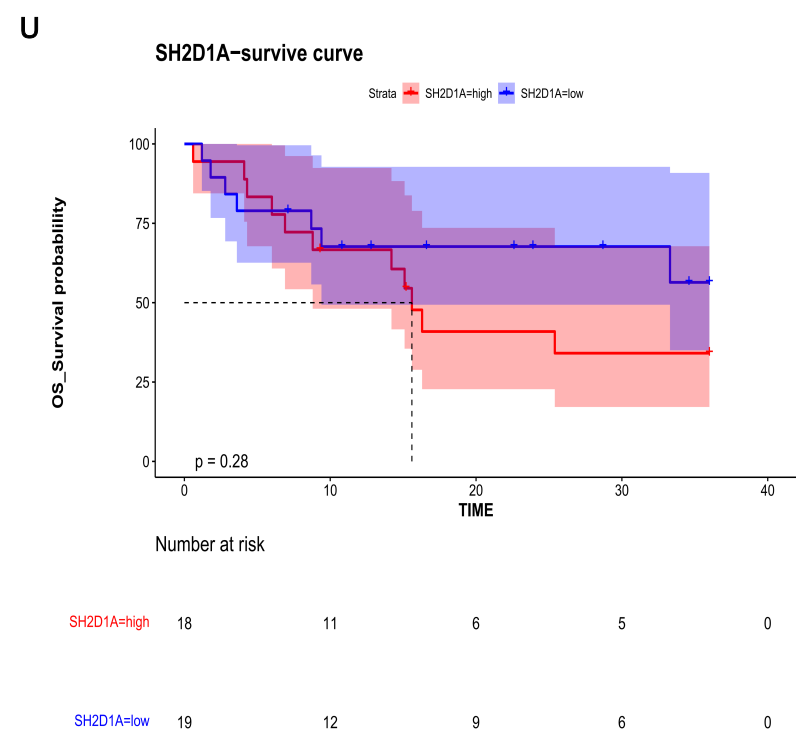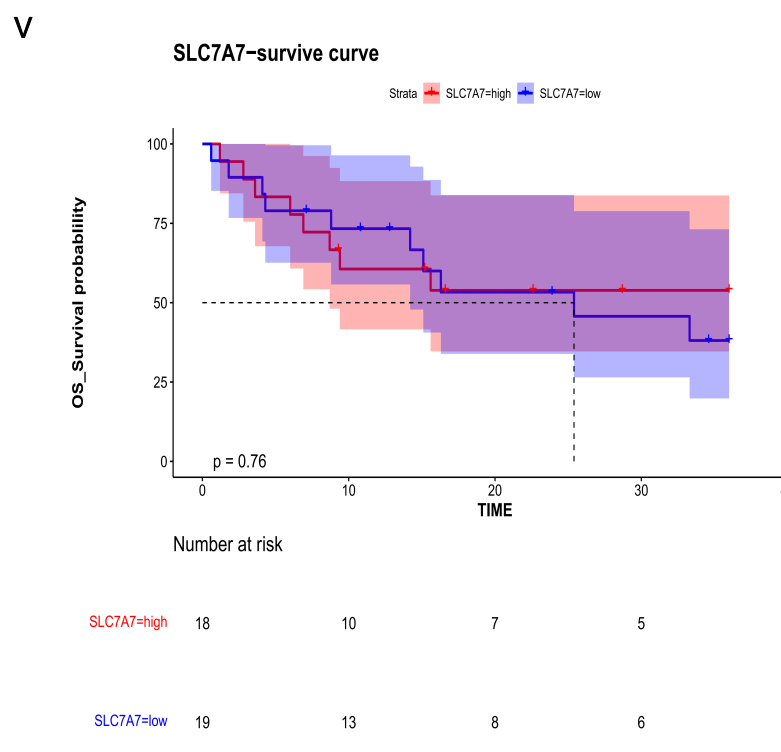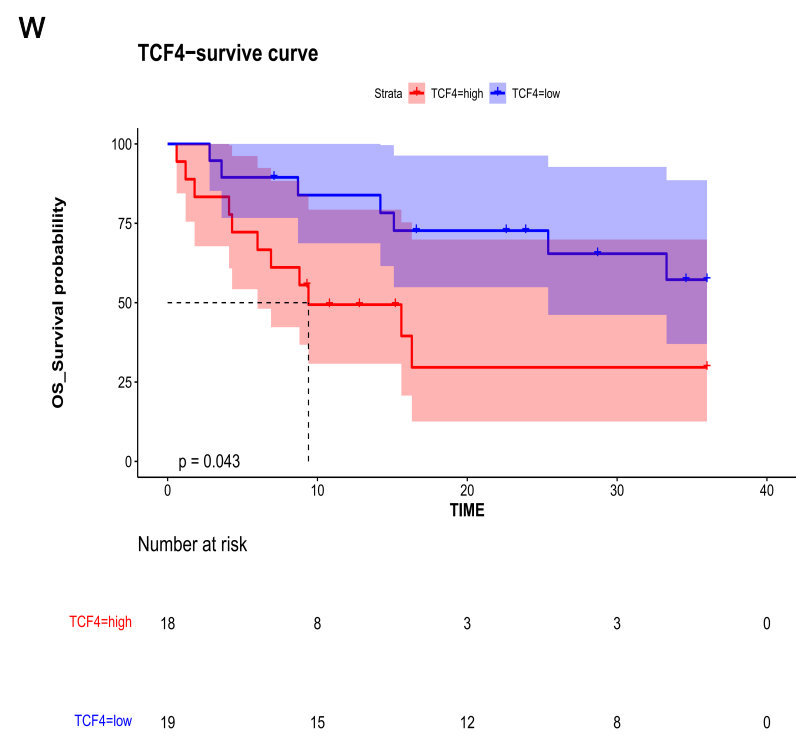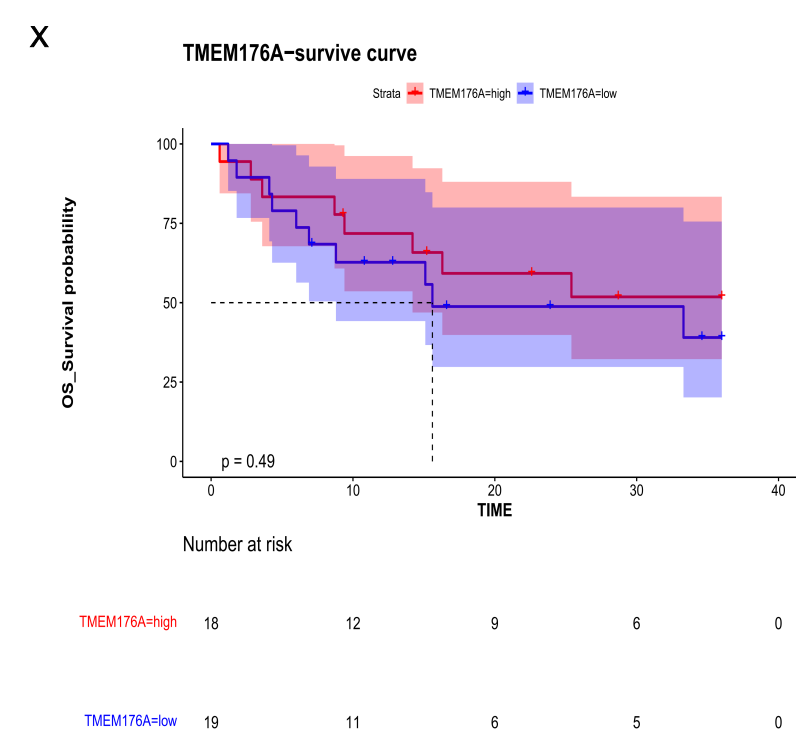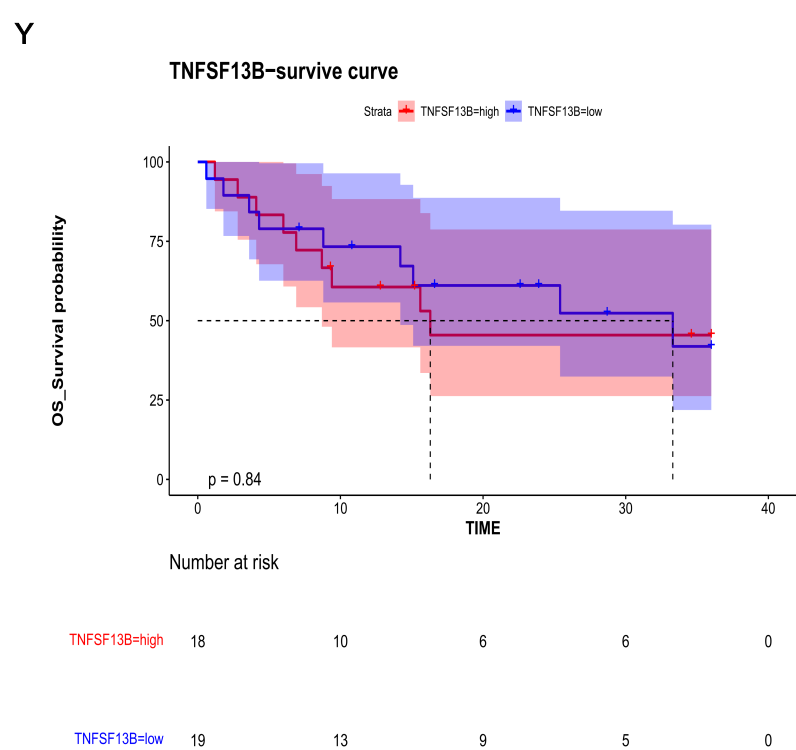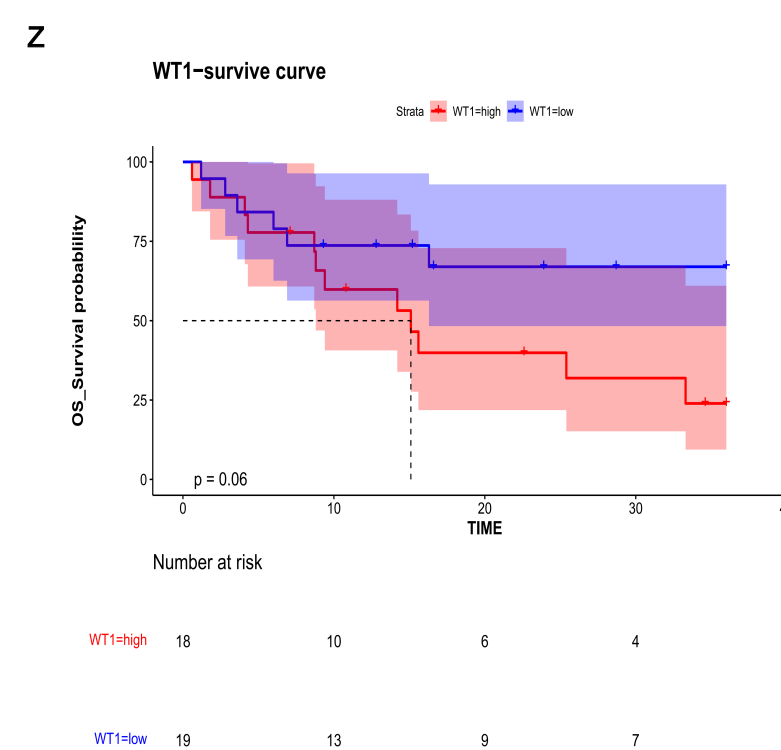

Supplement: Supplementary file 2 — Figure S2. Survival analysis of 26 risk genes in the validation cohort. (A) ABHD13; (B) BCKDK; (C) BTBD3; (D) CCL2; (E) CD1C; (F) CD300LF; (G) CTSD; (H) DCPS; (I) DGKE; (J) GCSAML; (K) GSTK1; (L) KCNK5; (M) MED12L; (N) METTL7B; (O) MSLN; (P) MT1X; (Q) N4BP3; (R) NUDT1; (S) OTUD6B; (T) SELL; (U) SH2D1A; (V) SLC7A7; (W) TCF4; (X) TMEM176A; (Y) TNFSF13B; (Z) WT1. [file CAM4-14-e70716-s002.pdf]
